# Defining and identifying cell sub-crosstalk pairs for characterizing cell–cell communication patterns

**DOI:** 10.1038/s41598-023-42883-8

**Published:** 2023-09-21

**Authors:** Chenxing Zhang, Yuxuan Hu, Lin Gao

**Affiliations:** School of Computer Science and Technology, Xidian University, Xi’an, 710071 China

**Keywords:** Cellular signalling networks, Computational models, Functional clustering

## Abstract

Current cell–cell communication analysis focuses on quantifying intercellular interactions at cell type level. In the tissue microenvironment, one type of cells could be divided into multiple cell subgroups that function differently and communicate with other cell types or subgroups via different ligand–receptor-mediated signaling pathways. Given two cell types, we define a cell sub-crosstalk pair (CSCP) as a combination of two cell subgroups with strong and similar intercellular crosstalk signals and identify CSCPs based on coupled non-negative matrix factorization. Using single-cell spatial transcriptomics data of mouse olfactory bulb and visual cortex, we find that cells of different types within CSCPs are significantly spatially closer with each other than those in the whole single-cell spatial map. To demonstrate the utility of CSCPs, we apply 13 cell–cell communication analysis methods to sampled single-cell transcriptomics datasets at CSCP level and reveal ligand–receptor interactions masked at cell type level. Furthermore, by analyzing single-cell transcriptomics data from 29 breast cancer patients with different immunotherapy responses, we find that CSCPs are useful predictive features to discriminate patients responding to anti-PD-1 therapy from non-responders. Taken together, partitioning a cell type pair into CSCPs enables fine-grained characterization of cell–cell communication in tissue and tumor microenvironments.

## Introduction

Cell–cell communication is primarily dependent on signal transduction pathways between cells. Signal-sending cells (sender cells) send regulatory signals to signal-receiving cells (receiver cells) mainly through ligand–receptor interactions, resulting in changes of states of the receiver cells^1,2^. In recent years, single-cell RNA-sequencing (scRNA-seq) technology enables comprehensive and systematic studies of cell–cell communication at unprecedented resolution^3^. Using scRNA-seq data, many computational tools have been developed to infer communication patterns between sender and receiver cell types, including activated ligand–receptor pairs (e.g. CellPhoneDB^4^ and CellChat^5^) or upstream and downstream signaling pathways (e.g. CytoTalk^6^ and scMLnet^7^). Combining scRNA-seq and CRISPR, Cohen et al. find a novel signaling pathway for communication between AT2 cells and basophils through IL33-IL1RL1 by comparing interleukin (IL)-33 receptor knockout and wild-type scRNA-seq data^8^; Schmidt et al. combine CRISPRa perturbations and single-cell RNA sequencing (CRISPRa Perturb-seq) to observe changes in downstream signaling pathways before and after T cell receptors activation^9^. For computational methods, Pirkl and Beerenwinkel design the mixture of Nested Effects Models to effectively infer the activated downstream signaling pathway in the single-cell transcriptome data (CROP-seq) of T-cell receptor activation^10^.

In fact, one cell type could be divided into multiple cell subgroups that function differently in tissue and tumor microenvironments^11–14^. For instance, Haim et al. find that astrocytes exhibit diverse morphological and physiological properties in brain tissues^15^. Zeisel et al. find many subtypes of astrocytes in the mouse nervous system, such as Müller glia and Bergmann glia^16^. Van der Leun et al. find that CD8 + T cells exhibit naïve-like, activated and exhausted states in tumor microenvironments^17^. Zemmour et al. study different activation states of T conventional cells and T regulatory cells^18^. For a sender-receiver cell type pair, cells can be partitioned into multiple subgroups with different ligand–receptor-mediated crosstalk signals. For example, Rodríguez-Ubreva et al. analyze the communication among B cell subtypes (e.g. naïve B cells and memory B cells), T cell subtypes (e.g. CD4 T cells and CD8 T cells), NK cell subtypes (e.g. NK16 cells and NK56 cells). They find significant differences in the inferred ligand–receptor pairs between cell subtypes, and some ligands or receptors are expressed in only a small number of cells in the subtypes^19^. Crouch et al. infer cell–cell communication between endothelia subtypes and mural subtypes in prenatal human brain. They find that midkine (MDK) expressed in most subtypes of endothelial (e.g. venous, capillary, and arterial subtypes) and mural cells (e.g. classic pericytes, fibroblasts, and smooth muscle cells), but syndecan 2 (SDC2, a receptor of MDK) is only weakly expressed in smooth muscle cells^20^.

Since the methods for inferring ligand–receptor pairs require the calculation of average gene expression for ligands or receptors in sender or receiver cells, this process weakens the subgroup-specific signal and preserves the conserved signal across multiple subgroups. These inferred ligand–receptor pairs between sender and receiver cell types likely consist of a mixture of different crosstalk signals originating from multiple subgroups. Therefore, it is a critical need to partition sender and receiver cell types into cell subgroups based on intercellular crosstalk signals. This partitioning will facilitate dissection of the mixed communication patterns between cell types from a fine-grained perspective. For a given sender-receiver cell type pair, we define a cell sub-crosstalk pair (CSCP) as a combination of a sender and receiver cell subgroups where cells in the sender cell subgroup communicate with cells in the receiver cell subgroup through similar activated ligand–receptor-mediated signaling pathways. We formulate the identification of CSCPs as a coupled non-negative matrix factorization (coupled NMF) problem (The derivation process is in Materials and Methods) which simultaneously divide two matrices (i.e. scRNA-seq data of sender and receiver cell type) based on the relationship between the features (e.g. known ligand–receptor interaction) of the two matrices.

To validate the effectiveness of CSCP in characterizing cell–cell communication, we focus on validating the results from three aspects: (1) spatial distance, (2) ligand–receptor pairs inference and (3) cancer immunotherapy prediction. Using single-cell spatial transcriptomics data, we find that the distances between sender cells and receiver cells within CSCPs are significantly smaller than those between cell types distributed in the whole spatial images, validating that CSCPs can effectively characterize local cell–cell communication. Constructing sampled scRNA-seq datasets with different signal-weakened ligand–receptor pairs from the same dataset, we apply 13 cell–cell communication inference methods to sampled scRNA-seq datasets at CSCP level. As the result, the ligand–receptor pairs identified within CSCPs have higher similarity across sampled data, validating that CSCPs facilitate revealing masked ligand–receptor pairs between cell types. Using scRNA-seq data from 29 breast cancer patients with different immunotherapy responses, we find higher predictive accuracy based on CSCPs, validating that the CSCPs are useful predictive features to distinguish between patients responding to anti-PD-1 therapy and non-responders. In addition, a Python package for identifying CSCPs is available on GitHub (https://github.com/GaoLabXDU/cell-sub-crosstalk-pair).

## Results

### Overview for defining and identifying cell sub-crosstalk pairs

**Figure 1:**
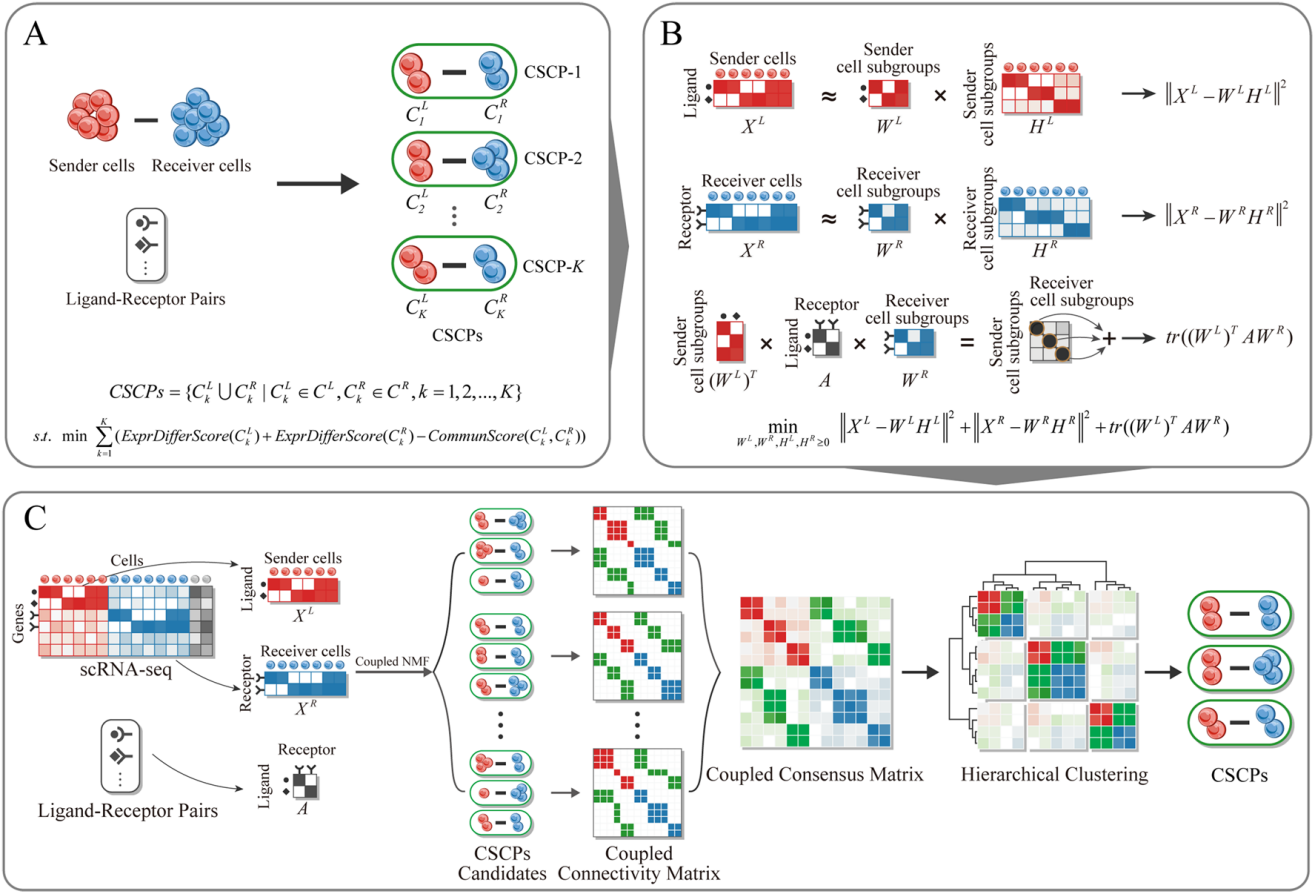
Definition and identification of CSCPs. (**A**) Defining CSCPs based on expression difference scores and communication scores, where expression difference score is designed to quantify the likelihood that the cells are functionally similar and communication score is designed to quantify the likelihood that the crosstalk signals mediated by ligand–receptor pairs are activated. (**B**) Identifying CSCPs by solving coupled non-negative matrix factorization (coupled NMF), which simultaneously decomposes the expression matrices of sender and receiver cell type under the constraints of known ligand–receptor pairs. (**C**) Using coupled consensus clustering to merge candidates of CSCPs, including constructing coupled connectivity matrix, constructing coupled consensus matrix and hierarchical clustering.

A CSCP is defined as a combination of a sender and receiver cell subgroups with strong and similar intercellular crosstalk signals. Within a CSCP, cells in the sender and receiver cell subgroups express similar ligand and receptor genes, respectively, indicating a likely communicate between them through similar ligand–receptor-mediated signaling pathways. Further, we simplify the ligand–receptor-mediated signaling pathway to the ligand–receptor interaction, as the latter serves as a bridge that connects two intracellular signaling pathways in ligand–receptor-mediated signaling pathways and is more important for cell–cell communication. Based on the definition and simplification mentioned above, we design an expression difference score to quantify the differences of cells based on ligand or receptor expression, and design a communication score to quantify the likelihood of activation of crosstalk signals mediated by ligand–receptor pairs (Fig. 1A). The identification of CSCPs involves dividing sender and receiver cell types into sender and receiver cell subgroups with low expression difference scores and high communication scores between them. This optimization problem is shown to be equivalent to the coupled non-negative matrix factorization (coupled NMF^21^) problem, where the objective of coupled NMF is to obtain an optimal decomposition of two matrices (i.e. scRNA-seq matrix of sender and receiver cell type) simultaneously under the constraints of the relationship between the features of two matrices (i.e. known ligand–receptor pairs) (Fig. 1B). To stabilize the results of coupled NMF, the coupled NMF algorithm is executed multiple times under different initializations to obtain multiple candidates of CSCPs. Subsequently, the coupled consensus clustering method is introduced to merge these candidates (Fig. 1C).

As the crucial factors for defining CSCPs, both ligand–receptor information and scRNA-seq data could impact identification of CSCPs. Ligand–receptor information is a part of the communication score definition, and its sensitivity needs to be considered. We apply two types of perturbation to the ligand–receptor pairs, that is, random deletion and random rematch. Subsequently, we quantify the similarity between the identified CSCPs based on perturbed and original ligand–receptor pairs (Fig. S1). As the result, we observe a decrease in similarity as the proportion of perturbation increases, suggesting that the identification of CSCPs is sensitive to changes in ligand–receptor pairs. The impact of random rematch on identifying CSCPs is greater than that of random deletion, indicating that CSCPs identification is more sensitive to errors in ligand–receptor information than to the absence of ligand–receptor information. The sparsity and noise within scRNA-seq data can obscure valuable information. In order to effectively identify CSCPs from sparse and noisy data, it is necessary to reduce the complexity of the method and narrow the range of feasible solutions, which requires an effective regularization of the method. Therefore, when using coupled NMF to identify CSCPs, we choose the commonly used L2 regularization to constrain the results of data decomposition^22^, thereby reducing the impact of data sparsity and noise and improving the stability of the results. To compare the stability between coupled NMF with and without L2 regularization, we calculate the similarity between the results of multiple runs. Figure S2 show that coupled NMF with L2 regularization exhibits higher similarity, indicating that L2 regularization can significantly improve the stability of coupled NMF.

### Cell sub-crosstalk pairs can effectively characterize local cell–cell communication

In the tissue microenvironment, the spatial distance between cells is closely related to the likelihood of communication between them, that is, cells in close proximity are more inclined to communicate^23,24^. This phenomenon serves as common criterion to validate the effectiveness of methods related to cell–cell communication. For example, Hu et al. introduce CytoTalk to infer inter-cellular network and compare the mutual information of expression of inferred ligand–receptor pairs in close cells with that in distant cells to verify the performance of their method^6^. Liu et al. systematically assess cell–cell communication methods by whether they can infer “short-range” ligand–receptor pairs in cells that are close to each other^25^. Therefore, the spatial distance between cells can serve as relatively dependable measure to validate the effectiveness of CSCPs.

**Figure 2:**
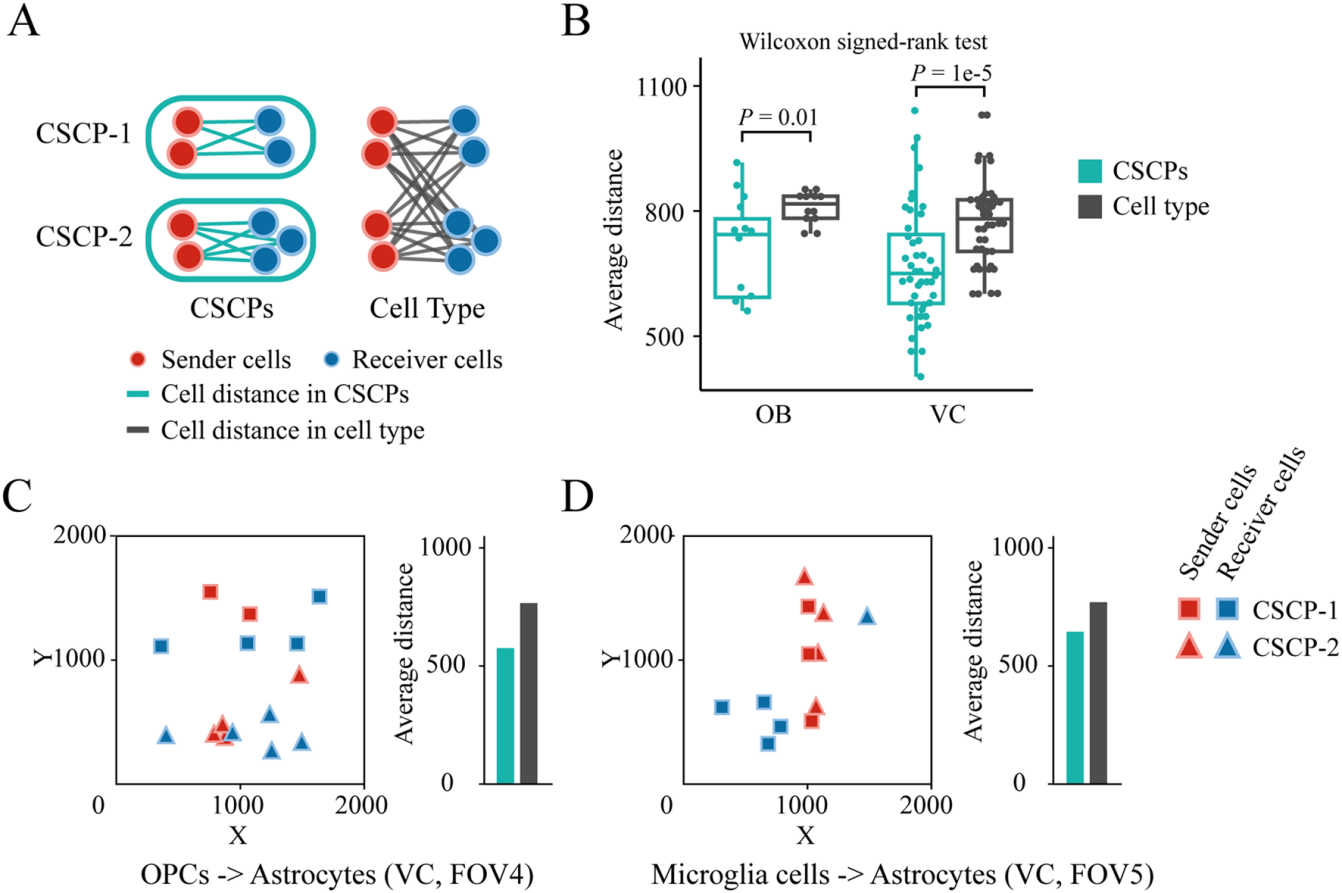
Spatial distance between cells in CSCPs and cell types. (**A**) Calculation of spatial distance between cells in CSCPs and cell type. At CSCP level, only cell pairs in the same CSCP between two cell types are considered. At cell type level, all cell pairs between two cell types are considered. (**B**) The box plot of cell distance between CSCPs and cell types. Each point represents the average distance between cells in each pair of cell types (black point) or corresponding CSCPs (green point) in each FOV. (**C**, **D**) Visualization of cell spatial location and corresponding bar plot of average distance between cells. The red squares represent the sender cells in CSCP-1, the blue squares represent the receiver cells in CSCP-1, the red triangles represent the sender cells in CSCP-2, the blue triangles represent the receiver cells in CSCP-2. *OB* olfactory bulb, *VC* visual cortex.

Based on the spatial distance between cells, we ask whether CSCPs can capture spatial patterns of cell–cell communication. To this end, we analyze sequential fluorescence in situ hybridization (seqFISH+) datasets of mouse olfactory bulb (OB) and visual cortex (VC). Both datasets contain seven fields of view (FOVs) on different locations in the same tissue. Each FOV covers five cell types that are known to communicate with each other^26–34^, including astrocytes, microglia, endothelial cells, oligodendrocytes and oligodendrocyte precursor cells (OPCs). We identify CSCPs for each of the 20 pairs of these cell types (sender cell type and receiver cell type are any two different cell types among these five cell types). Then, we calculate the average distance between cells of different types in CSCPs and the whole FOVs, respectively (Fig. 2A), and find that cells within the CSCPs are significantly closer with each other than in the whole FOVs in the olfactory bulb (P-value = 0.01 by Wilcoxon signed-rank test, Figs. 2B and S3A) and visual cortex (P-value = 1e−5 by Wilcoxon signed-rank test, Figs. 2B and S3B).

Furthermore, the visualization of single-cell spatial maps of the two tissues reveals that cells in the same CSCP are spatially distributed in localized cellular neighborhoods. For instance, two CSCPs containing OPCs and astrocytes are distributed in the upper and bottom cellular neighborhoods, respectively (Fig. 2C). Similarly, two CSCPs containing microglia and astrocytes are distributed in the left and right cellular neighborhoods, respectively (Fig. 2D). The visualization of microglia cells and endothelial cells in multiple FOVs of OB and VC datasets also exhibits same phenomenon. Some cells in CSCP tend to gather locally. For example, in FOV0 of OB, most of the cells in CSCP-1 tend to locate in the lower right corner, while most cells in CSCP-2 tend to locate in the upper left corner; in FOV4 of OB, most of the cells in CSCP-1 tend to locate at the left and lower side, while those in CSCP-2 tend to locate at the middle and upper side (Fig. S4).

Taken together, cells of different types within CSCPs have significantly smaller distances compared to those in the whole spatial map. This observation suggests that a CSCP is a group of cells with strong intercellular crosstalk signals, which can effectively characterize local cell–cell communication in the tissue microenvironment.

### Cell sub-crosstalk pairs can reveal ligand–receptor pairs masked at cell type level

Based on inferred ligand–receptor pairs using cell–cell communication methods, we validate that CSCP can effectively characterize cell–cell communication at a fine-grained level, revealing ligand–receptor pairs masked at cell type level. In fact, a single cell type could be further divided into multiple cell subgroups with distinct function^11^. Given a sender-receiver cell type pair, cells can be partitioned into multiple subgroups with different ligand–receptor-mediated crosstalk signals, some weak signals between cell subgroups could be masked at cell type level. For example, Rodríguez-Ubreva et al. analysis the intercellular communication between various subtypes of B cells, T cells, and NK cells. They explore the interactions between subtypes such as naïve B cells and memory B cells, CD4 T cells and CD8 T cells, as well as NK16 cells and NK56 cells. Through their analysis, they observe that certain ligands or receptors were only expressed in a limited number of cells within these subtypes^19^. Crouch et al. investigate the intercellular communication between different subtypes of endothelial and mural cells in the prenatal human brain. Their study reveals that midkine (MDK) is expressed in various endothelial subtypes, including venous, capillary, and arterial subtypes, as well as in various mural cells such as classic pericytes, fibroblasts, and smooth muscle cells. However, the expression of syndecan 2 (SDC2), which acts as a receptor for MDK, is only weakly expressed in smooth muscle cells^20^. Most existing cell–cell communication analysis methods infer activated ligand–receptor pairs at cell type level. Specifically, average expression of ligand and receptor genes in two given cell types indicates the activation degree of the ligand–receptor interactions, resulting in “mixed” communication signals detected. Therefore, the ability to reveal ligand–receptor pairs masked at the cell-type level can be used as a necessary condition for effectively characterizing cell–cell communication at a fine-grained level.

**Figure 3:**
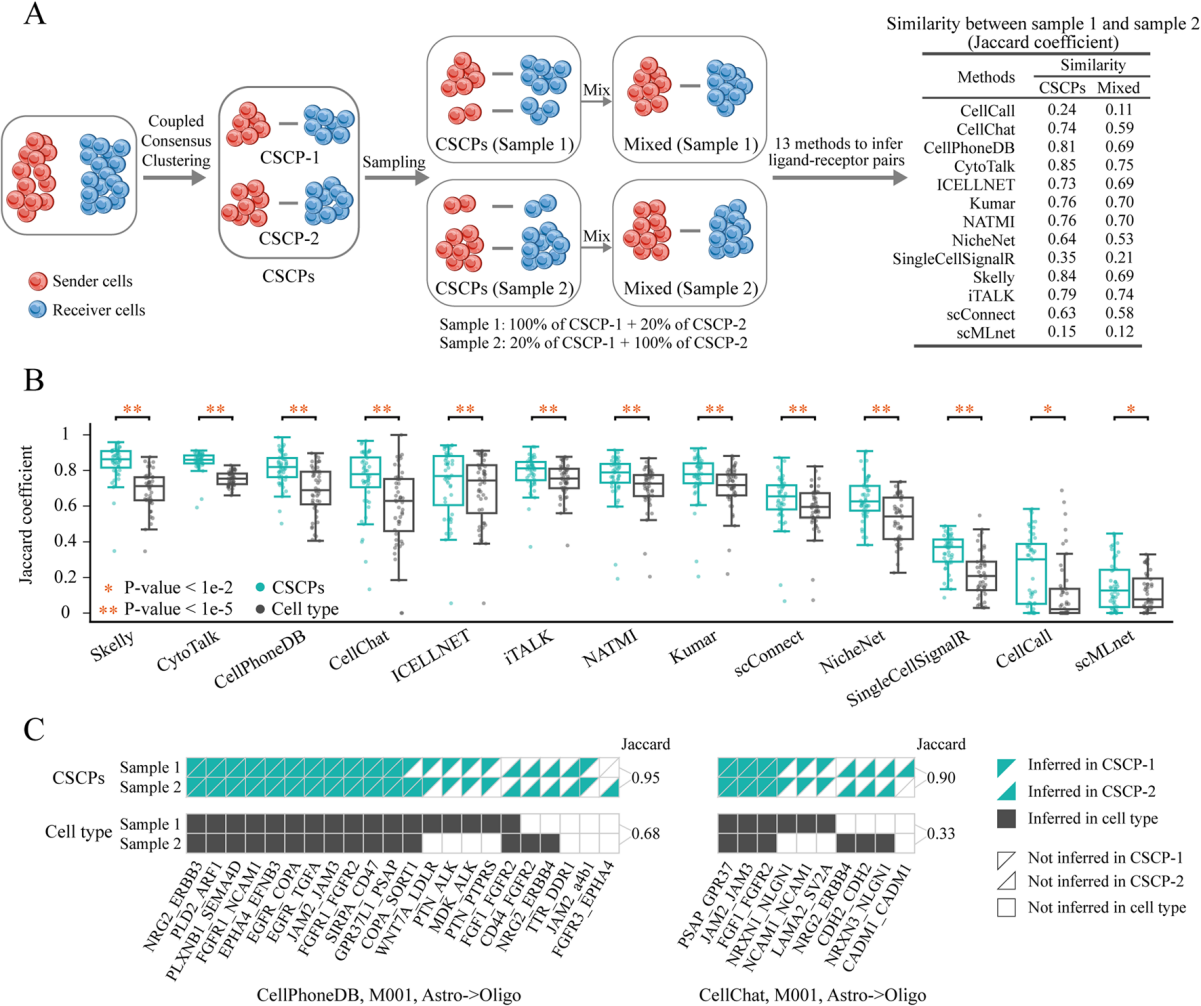
Similarity between inferred ligand–receptor pairs in sampled data. (**A**) Framework for constructing sampled data and quantifying the similarity between ligand–receptor pairs, including (1) two sampled data from each single-cell sequencing data (each cell type pair in each mouse) which masks a fraction of ligand–receptor pairs, (2) 13 state-of-art cell–cell communication methods for inferring ligand–receptor pairs, and (3) the Jaccard coefficient for quantifying the similarity between ligand–receptor pairs of the two sampled data. (**B**) Box plot of Jaccard coefficient. Each dot represents the Jaccard coefficient of ligand–receptor pairs between two sampled data of each cell type pair in each mouse. (**C**) Visualization of ligand–receptor pairs inferred by CellPhoneDB and CellChat. The green triangles indicate inferred ligand–receptor pairs in CSCPs and the black squares indicate inferred ligand–receptor pairs in cell types.

To demonstrate the effectiveness of CSCPs in revealing masked ligand–receptor pairs that cannot be identified at cell type level, we analyze scRNA-seq data on the cerebral cortex of four mice (two male and two female). Each data covers four cell types known to communicate with each other, including astrocytes, endothelial cells, microglia and oligodendrocytes^35^, which are combined into 12 cell type pairs. We sample the cells in each cell type pair for each mouse using the following two steps: (1) identifying two CSCPs (CSCP-1 and CSCP-2) for each cell type pair, and (2) randomly sampling cells in proportion to construct two sampled data (sample 1: keeping all cells in the CSCP-1 and randomly sampling 20% of the cells in the CSCP-2, sample 2: randomly sampling 20% of the cells in the CSCP-1 and keeping all cells in the CSCP-2). Considering the minimum cell proportion requirement (20%) for inferred ligand–receptor pairs established by Kumar et al.^36^, CSCP-1-specific ligand–receptor pairs in sample 2 and CSCP-2-specific ligand–receptor pairs in sample 1 would not be inferred at cell type level. Subsequently, we apply 13 cell–cell communication analysis methods (selected based on two criteria detailed in “Materials and methods”) to the sampled data. We then use the Jaccard coefficient to quantify the similarity between the inferred ligand–receptor pairs from sample 1 and sample 2 (Fig. 3A). We find that the similarity at CSCP level consistently outperforms than that at cell type level across all methods (Wilcoxon signed-rank test P-values < 0.01, Figs. 3B and S5–S8).

CellPhoneDB and CellChat are both representative cell–cell communication inference methods. They can infer not only the pairing of a single ligand with a single receptor but also the pairing of a single ligand with a complex composed of multiple receptors, which is consistent with biological facts. Moreover, both methods infer ligand–receptor pairs by calculating their statistical significance, reducing the false positives of inference. Both methods show that the similar ligand–receptor pairs could be inferred across two sampled data at CSCP level, but not at cell type level. Specifically, applying CellPhoneDB to oligodendrocytes and astrocytes, we find that the similarity of inferred ligand–receptor pairs in CSCPs is higher than that in cell types (Jaccard = 0.95 at CSCP level and 0.68 at cell type level, Fig. 3C). The similar phenomenon is observed when using CellChat to oligodendrocytes and astrocytes (Jaccard = 0.90 at CSCP level and 0.33 at cell type level, Fig. 3C) and when using CellPhoneDB to endothelial cells and oligodendrocytes (Jaccard = 0.95 at CSCP level and 0.47 at cell type level, Fig. S9). Meanwhile, we find that most of the ligand–receptor pairs shared by the two CSCPs are identified in both sampled data at either CSCP or cell type levels, such as NRG2-REBB3, PLD2-ARF1 and PLXNB1-SEMA4D when using CellPhoneDB for oligodendrocytes and astrocytes, PSAP-GPR37, JAM2-JAM3 and FGF1-FGFR2 when using CellChat for oligodendrocytes and astrocytes (Fig. 3C), and VTN-aVb1, EGFT-TGFA, EGFR-COPA, PROS1-TYRO3 and FGFR1-FGFR2 when using CellPhoneDB for endothelial cells and oligodendrocytes (Fig. S9). However, most CSCP-1-specific or CSCP-2-specific ligand–receptor pairs are identified in both sampled data at CSCP level only. At cell type level, these pairs are predicted in only one sampled data or even in neither sampled data. For instance, using CellPhoneDB to infer ligand–receptor pairs between oligodendrocytes and astrocytes, some CSCP-1-specific ligand–receptor pairs in sample 2 (e.g. WNT7A-LDLR, PTN-ALK, MDK-ALK and PTN-PTPRS) and CSCP-2-specific ligand–receptor pairs in sample 1 (e.g. CD44-FGFR2 and NRG2-ERBB4) are not identified at cell type level. When applying CellChat to oligodendrocytes and astrocytes, several CSCP-1-specific ligand–receptor pairs in sample 2 (e.g. NRXN1-NLGN1, NCAM1-NCAM1 and LAMA2-SV2A) and CSCP-2-specific ligand–receptor pairs in sample 1 (e.g. NRG2-ERBB4 and NRXN3-NLGN1) are not predicted at cell type level (Fig. 3C). When using CellPhoneDB to infer ligand–receptor pairs between endothelial cells and oligodendrocytes, some CSCP-1-specific ligand–receptor pairs in sample 2 (e.g. IGF2-IDE, IGF2-IGF1R, PLXNB2-SEMA4D, SEMA5A-PLXNB3) and CSCP-2-specific ligand–receptor pairs in sample 1 (e.g. ADORA2A-NAMPT, COPA-SORT1 and PTN-ALK) are not identified at cell type level (Fig. S9).

In addition, based on CSCPs, we infer some ligand–receptor pairs that are not inferred in both mixed datasets. For example, between astrocytes cells and oligodendrocytes cells, TTR-DDR1 is inferred to be a CSCP-2-specific ligand–receptor pair (Fig. 3C). Muntané et al. find that discoidin domain receptor tyrosine kinase 1 (DDR1) is known to be expressed in oligodendrocytes, and co-expressed with astrocyte-related genes to contribute to schizophrenia (SCZ) susceptibility^37^, and TTR is verified to be related to the energy metabolism of astrocyte^38^. Between endothelial cells and oligodendrocyte cells, NRP1-VEGFB is inferred to be a specific ligand receptor of CSCP-1 (Fig. S9). Sherafat et al. find that NRP1 and vascular endothelial cell growth factor (VEGF) in endothelial cells can affect oligodendrocyte regeneration^39^.

Together, inferring ligand–receptor pairs at CSCP level enables the discovery of masked cell–cell communication patterns at cell type level.

### Cell sub-crosstalk pairs are useful features to predict cancer immunotherapy response

Cancer immunotherapy has been approved to extend patient survival in many cancers, such as melanoma^40^, non-small cell lung cancer^41^ and breast cancer^42^. One of the most successful cancer immunotherapies is immune checkpoint blockade therapy, such as anti-PD-1, which blocks the intercellular communication between PD-L1 expressed on tumor cells or antigen presenting cells and PD-1 expressed on T cells, and reactivate the ability of T cells to identify and eliminate tumor cells. The expression of PD-L1 and PD-1 genes is an important indicator of the prognosis for anti-PD-1 therapy^43^. In fact, PD-L1–PD-1 is only activated among a small number of cell subgroups. For example, in tumor microenvironment of breast cancer, PD-1 is highly expressed in exhausted CD8 + T cells, and PD-L1 is highly expressed in macrophages (a cell subgroup of tumor-presenting cells)^42^. Therefore, the ability to predict response of cancer patients to anti-PD-1 therapy could be considered a clue to validate that CSCP can characterize cell–cell communication at a fine-grained level.

**Figure 4:**
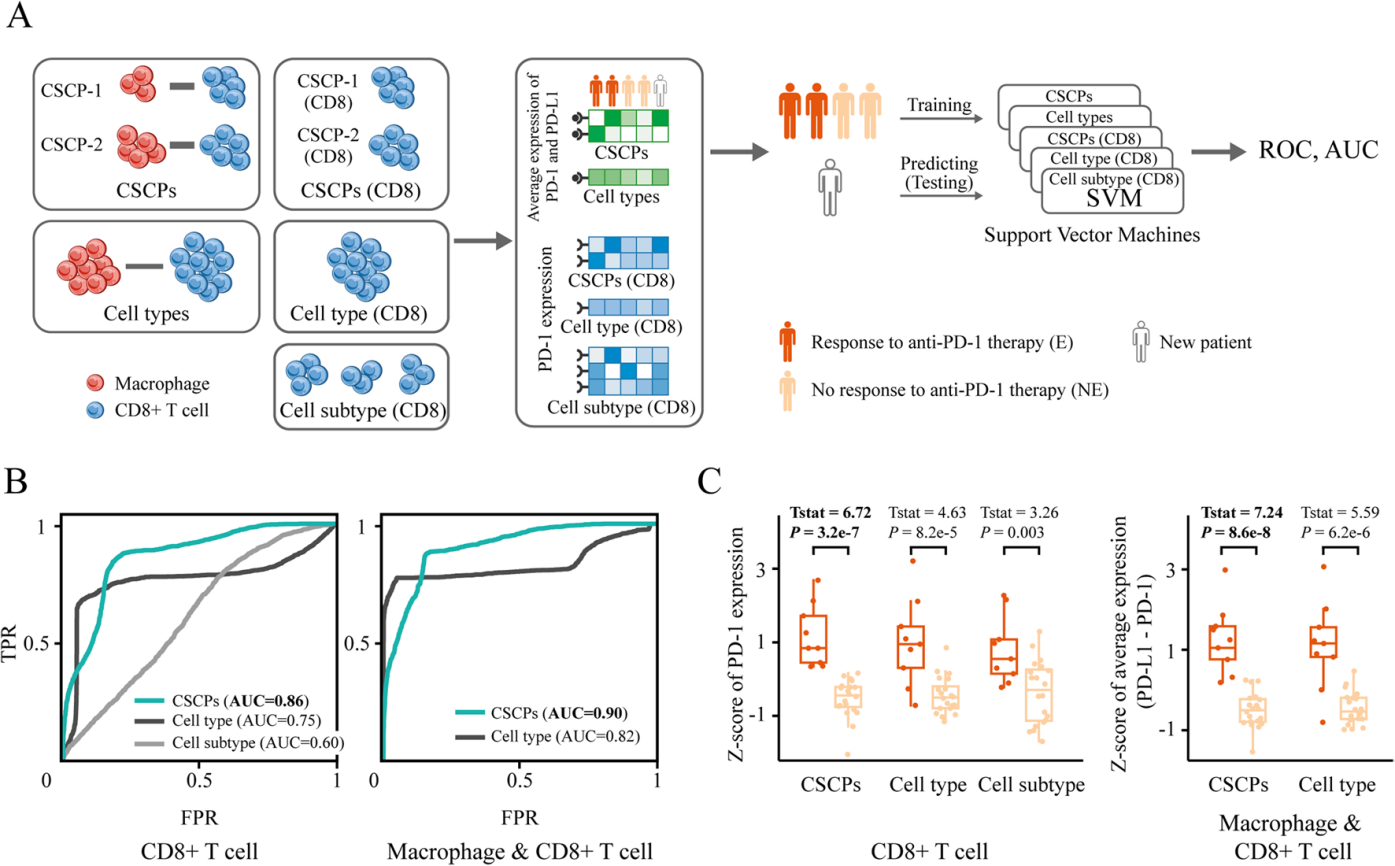
Prediction of anti-PD-1 therapy response based on CSCPs, cell type and cell subtype. (**A**) Diagram of prediction and comparison. For CSCPs and cell types, the predictive feature is the expression of PD-1 in CD8 + T cells, or the average expression of PD-1 and PD-L1 in CD8 + T cells and macrophage. For cell subtype, the predictive feature is the expression of PD-1 in each CD8 + T cell subtype. (**B**) Receiver operating characteristic curve (ROC) and area under curve (AUC) of predictive model based on CSCPs, cell type and cell subtype. (**C**) Box plot of normalized PD-1 expression or PD-L1–PD-1 average expression in CSCPs, cell type and cell subtype for each patient. Dark red indicates that the patients is responding to anti-PD-1 therapy and light red indicates that the patient is not responding to anti-PD-1 therapy.

We collect scRNA-seq data from tumor tissues of 29 breast cancer patients (20 non-responding patients and 9 responding patients) before anti-PD1 therapy^42^. According to the previous study^42,44^, anti-PD1 therapy is likely to target the interaction between PD-L1 on macrophages and PD-1 on CD8 + T cells because these two proteins are highly expressed in macrophages and CD8 + T cells, respectively, compared to other cell types in the tumor tissues. Therefore, we consider macrophage as the sender cell type and CD8 + T cell as receiver cell type, identifying two CSCPs for this cell type pair. To predict the patient response to the therapy, we first consider two types of features, including (1) PD-1 expression in all CD8 + T cells in the dataset, CD8 + T cell subtype and CD8 + T cells in the identified CSCPs and (2) average expression of PD-L1 and PD-1 in macrophages and CD8 + T cell and that in the corresponding CSCPs. Then, we employ a support vector machine for training, leveraging each of the features to predict patient therapy responses. The prediction accuracy is quantified by calculating the area under the receiver operating characteristic curve (AUC) which is drawn based on the true positive rate (TPR) of the model at different false positive rates (FPR), which are obtained by comparing the predicted results with the real results (Fig. 4A).

Comparing the AUC based on CD8 + T cells, we observe that the prediction model based on PD-1 expression in the CSCPs has the highest AUC of 0.86, while the AUC based on cell type and cell subtype are 0.75 and 0.60, respectively (Fig. 4B). Comparing the AUC based on CD8 + T cell and macrophage, the CSCP-based model still achieves the highest AUC of 0.90, while the cell-type-based is 0.82 (Fig. 4B). However, we are unable to obtain prediction result at cell subtype level due to the lack of matching information between macrophage subtypes and CD8 + T cell subtypes (i.e. which macrophage subtypes and which CD8 + T cell subtypes could communicate).

To further explain why the predictive model based on CSCPs is useful, we analyze the difference in the PD-1 expression or average expression of PD-L1 and PD-1 between non-responding patients and responding patients. We first perform dimensionality reduction (linear discriminant analysis, LDA) and normalization (z-score normalization) of PD-1 expression and average expression of PD-L1 and PD-1. This ensures that the observed differences are comparable across CSCPs, cell type and cell subtype. Specifically, we condense the expression data from cell subtype and CSCPs into a one-dimensional expression for each patient, and then normalize the one-dimensional expression in cell type, cell subtype and CSCPs across all patients respectively (the schematic diagram of dimensionality reduction and normalization is shown in Fig. S10 and the dimensionality-reduced and normalized expressions of each feature in each patient are shown in Fig. S11). Then, using t-statistics and t-test to quantify the differences and their significance in PD-1 expression and the average expression of PD-L1 and PD-1 between responding and non-responding patients, we observe that the normalized expression in CSCPs has the largest difference and most significant (t-statistic = 6.72 and P-value = 3.2e−7 of PD-1 expression, t-statistic = 7.24 and P-value = 8.6e−8 of average expression of PD-L1 and PD-1, Fig. 4C).

Together, CSCPs are useful predictive features to distinguish patients responding to anti-PD-1 therapy from non-responders.

## Discussion

CSCPs are useful for effectively characterizing cell–cell communication in local cellular neighborhoods, providing a fine-grained view to reveal intercellular crosstalk signals in tissue and tumor microenvironments.

Identification of CSCPs relies on data-supported and literature-supported interactions between cell types. When validating CSCPs using cancer immunotherapy prediction, we identify CSCPs between macrophages and CD8 + T cells as predictive features, because the interaction between these two cell types is supported by both data and literature. For instance, Bassez et al. find that PD-L1 is highly expressed in tumor-associated macrophages, and PD-1 is highly expressed in exhausted CD8 + T cells^42^. Petty et al. demonstrate that tumor-associated macrophages bind to CD8 + T cells through PD-L1 and PD-1 interactions, inhibiting the activity of CD8 + T cells and reducing tumor immune responses^14^. It is important to emphasize that CSCPs could not be identified in the absence of matching information. For example, identifying CSCPs between macrophage subtypes and CD8 + T cell subtypes requires corresponding data and literature to support information regarding which macrophage subtypes match with which CD8 + T cell subtypes.

While coupled NMF is more suitable for CSCP identification due to its non-negativity and interpretability, alternative computational methods can also be considered, such as network analysis and machine learning. However, network analysis (e.g. graph-based clustering^45,46^) and machine learning (e.g. autoencoder-based methods^47,48^) require additional operations and constraints in terms of interpretability and non-negativity. For example, graph-based clustering needs to further identify expressed ligand or receptor genes in clustering results through differential expression methods, such as limma^49^ and edgeR^50^. The autoencoder-based methods should consider non-negativity constraints, such as NCAE^51^ and NCSAE^52^. For the classification of breast cancer patients with responding and non-responding to anti-PD-1 therapy, we use support vector machine (SVM) as classifier. Comparing with other methods such as XGBoost^53^ and random forest^54^, SVM can better reflect the advantages of CSCP-based features. Because the tree-boosting methods actually use new features that are further extracted during classifying, which could not reflect the advantages of the original features (i.e. CSCP-base features). Moreover, Fig. 4C illustrates the results of linear dimensionality reduction (LDA) of CSCP-based features and cell-type-based features, there is significant difference between the features of non-responding patients and responding patients. It indicates that there is significant decision plane (hyperplane) in the original feature space. In addition, we employ linear discriminant analysis (LDA) as a supervised and linear dimension reduction technique. Because LDA can find a dimension that distinguishes non-responding patients from responding patients as much as possible, allowing us to clearly compare the difference of gene expression between two types of patients on this dimension. Other dimensionality reduction methods, such as principal component analysis (PCA), multidimensional scaling (MDS), may not be able to find the dimension that optimally distinguishes the two types of patients, thus affecting the performance of comparing gene expression (Fig. S12).

By incorporating additional information, CSCP can enhance its ability to accurately characterize cell–cell communication, such as spatial information^55^, dynamic information^56^ and upstream and downstream signaling pathways of ligands and receptors^6,57^. Spatial information is crucial for charactering cell–cell communication. However, most spatial transcriptomic technologies are at the spot level, lacking the resolution required to study cell–cell communication at the single-cell level, such as ST^58^ and 10 × Visium^59^. Single-cell spatial proteomic technology provide proteomic data and location information at single-cell resolution, such as CODEX^55^. Nonetheless, it can only measure the abundance of a few dozen proteins, with only a small fraction of them being ligands and receptors. Therefore, current spatial data is insufficient for defining CSCPs. Once the spatial transcriptomic or proteomic technology of the whole transcriptome or proteome at the single-cell level is available, pre-dividing cell types into cell subpopulations according to the spatial position of cells in the spatial transcriptome or proteome can enable CSCPs to characterize local cell–cell communication in a more fine-grained perspective. Moreover, cell–cell communication in tissues or tumor microenvironments is a dynamic process, such as cellular differentiation and the immune response. This requires adding temporal dimension when studying intercellular communication^56^. For example, Anchang et al. present framework of dynamic spanning forest mixtures (DSFMix) and use temporal single-cell data at discrete time points to analyze the communication between somatic cells and differentiating cells during spermatogenesis^60^. Atitey et al. propose multiscale multicellular quantitative evaluator (MMQE), a hybrid ordinary differential equation-based (ODE) multicellular model system and analysis the effects of cell–cell communication based on IL-2 and IL-4 based on lymphocyte differentiation during immune responses^61^. Li et al. propose a computational method, called TraSig, to infer cell–cell interactions using single-cell pseudotime trajectories^62^. In order to enable CSCPs to characterize dynamic intercellular communication, it may be a feasible way to use the differentiation trajectory^63^ as a constraint of coupled NMF or consider dynamic network based non-negative matrix factorization^64^. In addition, the activated ligand–receptor-mediated signaling pathways are composed of the upstream signal pathway of the activated ligand in the sender cells, the ligand–receptor interaction, and the downstream signal pathway of the activated receptor in the receiver cells, which can confirm that communication occurs between sender cells and receiver cells. In the definition of CSCP, only ligand–receptor interaction is considered because it is a bridge connecting two intracellular signaling pathways and is more important for cell–cell communication. But the role of upstream and downstream signaling pathways in characterizing cell–cell communication cannot be ignored. Hu et al. construct intra-cellular and inter-cellular signaling network and perform a comparative analysis of signaling networks between macrophages and endothelial cells in human adult and fetal tissues^6^. To integrate network information, we can consider expression of genes in the ligand–receptor upstream and downstream pathways.

Experimentally verifying CSCP presents a challenge. One potential but time-consuming and expensive way involves combining CRISPR with scRNA-seq. This would require knocking out the inferred ligands or receptors within CSCPs' cells, followed by transcriptome sequencing using single-cell sequencing technology. By identifying differentially expressed genes between the experimental and wild groups and performing pathway (or functional) enrichment analysis, we can validate the association between CSCP and cell–cell communication. For instance, using single-cell RNA-seq data from IL1RL1 knockout mice, Cohen et al. find that AT2 cells and basophils communicate through IL33-IL1RL1 and its signaling pathways^8^. Additionally, Hu et al. employ differentially expressed genes between IL1RL1-knockout and wild-type basophils and functional enrichment analysis of the inferred communication network to validate the performance of their cell–cell communication method^6^. In addition, as shown in the results, the spatial distance of cells within CSCP can be used to validate the ability of CSCP to character cell–cell communication, because the spatial distance between cells is closely correlated with whether there is communication between them^23,24^. For example, Hu et al. compare the inferred communication pattern between close cells and distance cells to validate that their cell–cell communication method is effective^6^. Liu et al. define “short-range” and “long-range” ligand–receptor pairs to evaluate 16 cell–cell communication methods^25^.

Only two cell types are considered for one CSCP, which is the simplest and most ideal one-to-one cell–cell communication. In fact, in the real tissue or tumor microenvironment, the cell–cell communication is a many-to-many relationship, the communication between multiple cell types needs to be considered^65^. To characterize the many-to-many communication, we can split a many-to-many relationship into multiple one-to-one relationships, for example, communication between three cell types can be split into communication between three cell type pairs (do not distinguish sender and receiver cell type) or six cell type pairs (distinguish sender and receiver cell type). Then, we separately identify multiple one-to-one CSCPs and merge them into a many-to-many CSCPs.

In addition, coupled NMF has the potential to infer ligand–receptor pairs in CSCPs and is only used to identify CSCPs in this study. However, the focus of this study is to define CSCPs and illustrate that CSCPs provide a new perspective for studying cell–cell communication. Inferring and analyzing communication patterns in CSCPs will be the focus of our next work.

## Materials and methods

### Definition of the cell sub-crosstalk pairs

In the tissue microenvironment, cells of one type could perform different functions, implying that those cells express different proteins or genes. Therefore, the similarity of cell functions can be measured based on gene expression profiles. We sum the differences of expression profiles at the cell and gene levels, denoted as the expression difference score, to infer the likelihood that the cells are functionally similar. That is, lower expression difference score implies higher likelihood of functional similarity.

Cells with different functions in the sender and receiver cell types could express different ligand and receptor genes, respectively, resulting in different activated ligand–receptor-mediated signaling pathways for cell–cell communication. Specifically, those expressed ligand or receptor genes are further translated into proteins, and when the ligand protein of one cell binds to the corresponding receptor protein of another cell, ligand–receptor-mediated crosstalk signaling is activated. We quantify the likelihood of crosstalk signal activation mediated by a ligand–receptor pair by multiplying the expression of the ligand gene with that of the corresponding receptor gene, and define communication score as the sum of these likelihoods for all ligand–receptor pairs. The high communication score between two cell subgroups indicates a high probability of intercellular crosstalk between them.

We define a CSCP consisting of a subgroup of sender cells and a subgroup of receiver cells with strong crosstalk signals between them, where the cells in sender cell subgroup express the similar ligand genes, the cells in receiver cell subgroup express the similar receptor genes, and the cells in sender cell subgroup communicate with cells in receiver cell subgroup through multiple activated ligand–receptor-mediated signaling pathways. The CSCPs are unions of a sender cell subgroup and a receiver cell subgroup with low expression difference score and high communication score.

Given $K$ is the number of CSCPs, $C^{L} = \{ C_{1}^{L} ,C_{2}^{L} ,...,C_{K}^{L} \}$ and $C^{R} = \{ C_{1}^{R} ,C_{2}^{R} ,...,C_{K}^{R} \}$ is the partition of sender cells and receiver cells (i.e. the set of sender cell subgroups and receiver cell subgroups), respectively, the definition is shown in Fig. 1A and formulated below:

$$ CSCPs = \{ C_{k}^{L} \cup C_{k}^{R} |C_{k}^{L} \in C^{L} ,C_{k}^{R} \in C^{R} ,k = 1,2,...,K\} $$

$$ s.t.\begin{array}{*{20}c} {} \\ \end{array} \min \begin{array}{*{20}c} { \, \sum\limits_{k = 1}^{K} {(ExprDifferScore(C_{k}^{L} ) + ExprDifferScore(C_{k}^{R} ) - CommunScore(C_{k}^{L} ,C_{k}^{R} ))} } \\ \end{array} $$

where $ExprDifferScore$ calculate the difference between the expression of genes expressed in a cell subgroup and their average expression, and $CommunScore$ calculate the proportion of activated (expressed) ligand–receptor pairs between sender cell subgroup and receiver cell subgroup. The function of $ExprDifferScore$ and $CommunScore$ is as follows:

$$ ExprDifferScore(C_{k}^{L} ) = \sum\limits_{{c^{L} \in C_{k}^{L} }} {\left\| {{\varvec{x}}_{{c^{L} }} - {\varvec{m}}_{{C_{k}^{L} }} } \right\|^{2} } + \sum\limits_{{g^{L} \in G_{k}^{L} }} {\left\| {{\varvec{y}}_{{g^{L} }} - {\varvec{m}}_{{G_{k}^{L} }} } \right\|^{2} } $$

$$ ExprDifferScore(C_{k}^{R} ) = \sum\limits_{{c^{R} \in C_{k}^{R} }} {\left\| {{\varvec{x}}_{{c^{R} }} - {\varvec{m}}_{{C_{k}^{R} }} } \right\|^{2} } + \sum\limits_{{g^{R} \in G_{k}^{R} }} {\left\| {{\varvec{y}}_{{g^{R} }} - {\varvec{m}}_{{G_{k}^{R} }} } \right\|^{2} } $$

$$ CommunScore(C_{k}^{L} ,C_{k}^{R} ) = \sum\limits_{{g^{L} \in G_{k}^{L} }} {\sum\limits_{{g^{R} \in G_{k}^{R} }} {w_{{g^{L} ,k}}^{{}} a_{{g^{L} ,g^{R} }} w_{{g^{R} ,k}}^{{}} } } $$

where $G_{k}^{L}$ and $G_{k}^{R}$ is the expressed genes in $C_{k}^{L}$ and $C_{k}^{R}$, respectively. ${\varvec{x}}_{c}$ is the expression vector of a cell in all genes, ${\varvec{y}}_{g}$ is the expression vector of a gene in all cells. ${\varvec{m}}_{{C_{k}^{L} }}$ or ${\varvec{m}}_{{C_{k}^{R} }}$ is the average expression vector in each ligand gene or receptor gene of all cells in $C_{k}^{L}$ or $C_{k}^{R}$, respectively. ${\varvec{m}}_{{G_{k}^{L} }}$ or ${\varvec{m}}_{{G_{k}^{R} }}$ is the average expression vector in all ligand genes or receptor genes per cell in $G_{k}^{L}$ or $G_{k}^{R}$, respectively. $a_{{g^{L} ,g^{R} }}$ is the indicator of ligand–receptor pairs, $a_{{g^{L} ,g^{R} }} = 1$ if $g^{L} ,g^{R}$ is the matched ligand–receptor pair, otherwise 0. $w_{{g^{L} ,k}}$ and $w_{{g^{R} ,k}}$ represent the expression of $g^{L}$ and $g^{R}$ in $G_{k}^{L}$ and $G_{k}^{R}$, respectively.

### Identification of the cell sub-crosstalk pairs

A CSCP consists of a subgroup of sender cells and a subgroup of receiver cells with strong and similar intercellular crosstalk signals between them. This means that a CSCP consists of a sender cell subgroup and a receiver cell subgroup with low expression difference score and high communication score between them. Identifying CSCPs is to solve the following objective function:

$$ \mathop {\min }\limits_{{\{ C_{k}^{L} ,C_{k}^{R} |k = 1,2,...,K\} }} \begin{array}{*{20}c} { \, \sum\limits_{k = 1}^{K} {(ExprDifferScore(C_{k}^{L} ) + ExprDifferScore(C_{k}^{R} ) - CommunScore(C_{k}^{L} ,C_{k}^{R} ))} } \\ \end{array} $$

Expanding each item based on the definition of $ExprDifferScore$ and $CommunScore$:

$$ \mathop {\min }\limits_{{\{ C_{k}^{L} ,C_{k}^{R} |k = 1,2,...,K\} }} \sum\limits_{k = 1}^{K} {\left( {\sum\limits_{{c^{L} \in C_{k}^{L} }} {\left\| {{\varvec{x}}_{{c^{L} }} - {\varvec{m}}_{{C_{k}^{L} }} } \right\|^{2} + \sum\limits_{{g^{L} \in G_{k}^{L} }} {\left\| {{\varvec{y}}_{{g^{L} }} - {\varvec{m}}_{{G_{k}^{L} }} } \right\|^{2} } + \sum\limits_{{c^{L} \in C_{k}^{R} }} {\left\| {{\varvec{x}}_{{c^{R} }} - {\varvec{m}}_{{C_{k}^{R} }} } \right\|^{2} } + \sum\limits_{{g^{R} \in G_{k}^{R} }} {\left\| {{\varvec{y}}_{{g^{R} }} - {\varvec{m}}_{{G_{k}^{R} }} } \right\|^{2} } - \sum\limits_{{g^{L} \in G_{k}^{L} }} {\sum\limits_{{g^{R} \in G_{k}^{R} }} {w_{{g^{L} ,k}}^{{}} a_{{g^{L} ,g^{R} }} w_{{g^{R} ,k}}^{{}} } } } } \right)} $$

Combining the first and second items, and the third and fourth items, respectively, the following function is obtained:

$$ \mathop {\min }\limits_{{\{ C_{k}^{L} ,C_{k}^{R} |k = 1,2,...,K\} }} J_{1} + J_{2} - J_{3} $$

where

$$ J_{1} = \sum\limits_{k = 1}^{K} {\left( {\sum\limits_{{c^{L} \in C_{k}^{L} }} {\left\| {{\varvec{x}}_{{c^{L} }} - {\varvec{m}}_{{C_{k}^{L} }} } \right\|^{2} + \sum\limits_{{g^{L} \in G_{k}^{L} }} {\left\| {{\varvec{y}}_{{g^{L} }} - {\varvec{m}}_{{G_{k}^{L} }} } \right\|^{2} } } } \right)} $$

$$ J_{2} = \sum\limits_{k = 1}^{K} {\left( {\sum\limits_{{c^{R} \in C_{k}^{R} }} {\left\| {{\varvec{x}}_{{c^{R} }} - {\varvec{m}}_{{C_{k}^{R} }} } \right\|^{2} } + \sum\limits_{{g^{R} \in G_{k}^{R} }} {\left\| {{\varvec{y}}_{{g^{R} }} - {\varvec{m}}_{{G_{k}^{R} }} } \right\|^{2} } } \right)} $$

$$ J_{3} = \sum\limits_{k = 1}^{K} {\sum\limits_{{g^{L} \in G_{k}^{L} }} {\sum\limits_{{g^{R} \in G_{k}^{R} }} {w_{{g^{L} ,k}}^{{}} a_{{g^{L} ,g^{R} }} w_{{g^{R} ,k}}^{{}} } } } $$

According to the Ding’s proof^66^, there are the following equivalence relations:

$$ \mathop {\min }\limits_{{\{ C_{k}^{L} |k = 1,2,...,K\} }} J_{1} \Leftrightarrow \mathop {\min }\limits_{{W^{L} ,H^{L} \ge 0}} \, \left\| {X^{L} - W^{L} H^{L} } \right\|^{2} $$

$$ \mathop {\min }\limits_{{\{ C_{k}^{R} |k = 1,2,...,K\} }} J_{2} \Leftrightarrow \mathop {\min }\limits_{{W^{R} ,H^{R} \ge 0}} \, \left\| {X^{R} - W^{R} H^{R} } \right\|^{2} $$

where $X^{L}$ and $X^{R}$ is the expression matrix of sender cells and receiver cells, respectively. $H^{L}$ and $H^{R}$ is the matrix indicating which sender and receiver cells belong to which sender and receiver cell subgroup, respectively. $W^{L}$ and $W^{R}$ is the matrix indicating which ligand and receptor genes are expressed in which sender and receiver cell subgroup, respectively.

Meanwhile, $J_{3}$ can be described in the form of matrix calculation:

$$ J_{3} = tr((W^{L} )^{T} AW^{R} ) $$

where $A$ is the indication matrix of ligand–receptor gene pairs.

Therefore, the identification of CSCPs is transformed into solving the following optimization function (Fig. 1B):

$$ \mathop {\min }\limits_{{W^{L} ,W^{R} ,H^{L} ,H^{R} \ge 0}} \, \left\| {X^{L} - W^{L} H^{L} } \right\|^{2} + \left\| {X^{R} - W^{R} H^{R} } \right\|^{2} + tr((W^{L} )^{T} AW^{R} ) $$

This objective function is similar as that of coupled non-negative matrix factorization (coupled NMF)^21^, that is:

$$ \mathop {\min }\limits_{{W^{L} ,W^{R} ,H^{L} ,H^{R} \ge 0}} \frac{1}{2}\left\| {X^{L} - W^{L} H^{L} } \right\|_{F}^{2} + \frac{{\lambda_{1} }}{2}\left\| {X^{R} - W^{R} H^{R} } \right\|_{F}^{2} - \lambda_{2} tr((W^{L} )^{T} AW^{R} ) + \mu (\left\| {W^{L} } \right\|_{F}^{2} + \left\| {W^{R} } \right\|_{F}^{2} ) $$

where the additional parameters and regularization terms are used to balance the weights between the terms and constrain the sparsity of the results, respectively.

The multiplicative update rule is used to solve coupled NMF:

$$ w_{ij}^{L} \leftarrow w_{ij}^{L} \frac{{\left( {X^{L} (H^{L} )^{T} + \frac{{\lambda_{2} }}{2}A^{T} W^{R} } \right)_{ij} }}{{(W^{L} H^{L} (H^{L} )^{T} + 2\mu W^{L} )_{ij} }}\;\;\;\;\;\;\;\;w_{ij}^{R} \leftarrow w_{ij}^{R} \frac{{\left( {X^{R} (H^{R} )^{R} + \frac{{\lambda_{2} }}{{2\lambda_{1} }}AW^{L} } \right)_{ij} }}{{(W^{R} H^{R} (H^{R} )^{T} + 2\mu W^{R} )_{ij} }} $$

$$ h_{ij}^{L} \leftarrow h_{ij}^{L} \frac{{((W^{L} )^{T} X^{L} )_{ij} }}{{((W^{L} )^{T} W^{L} H^{L} )_{ij} }}\;\;\;\;\;\;\;\;h_{ij}^{R} \leftarrow h_{ij}^{R} \frac{{((W^{R} )^{T} X^{R} )_{ij} }}{{((W^{R} )^{T} W^{R} H^{R} )_{ij} }} $$

Since the method related to non-negative matrix factorization is influenced by initialization, that is, there is difference in the results under different initializations. To reduce this difference in identifying CSCPs, we repeatedly execute the coupled NMF with different initialization to obtain multiple candidates of CSCPs and propose coupled consensus clustering to merge these candidates (Fig. 1C), including constructing and clustering coupled consensus matrix. For constructing coupled consensus matrix, we first construct the coupled connectivity matrix $P^{t}$ for the $t$-th candidates (i.e. the result for the $t$-th execution of the method) according to the following rule:

$$ p_{i,j}^{t} = \left\{ {\begin{array}{*{20}l} {1,} \hfill & {if\; \, c_{i} ,c_{j} \in C_{k}^{L} \cup C_{k}^{R} , \, \;1 \le k \le K} \hfill \\ {0,} \hfill & {otherwise} \hfill \\ \end{array} } \right. $$

where $t$ represents that the $t$-th candidate of CSCPs ($T$ times in total).

Then, we merge the coupled connectivity matrix $P^{t}$ of $T$ candidates into a coupled consensus matrix and normalized it with $T$. Finally, we perform the hierarchical clustering (The "AgglomerativeClustering" function of the ‘sklearn’ Python package, with default parameters) on the coupled consensus matrix, obtaining the stable CSCPs.

### Initialization of coupled NMF

For the initialization of coupled NMF, we generate 4 non-negative random matrices with the same scale as $W^{L}$,$W^{R}$,$H^{L}$,$H^{R}$. For multiple runs of coupled NMF, we randomly generate different initialization matrices with different randomization seeds.

### Parameter selection

Coupled NMF contains four parameters, that is $\lambda_{1}$,$\lambda_{2}$,$\mu$ and $K$, where $\lambda_{1}$ controls the balance of the decomposition of $X^{L}$ and $X^{R}$. $\lambda_{2}$ controls the weight of communication score. $\mu$ control the weight of regularization term. $K$ is the number of CSCPs. The selection of $\lambda_{1}$, $\lambda_{2}$ and $\mu$ is the same as that of Duren et al.^21^:

$$ \lambda_{1} = \frac{{\left\| {X^{L} - W^{L} H^{L} } \right\|_{F}^{2} }}{{\left\| {X^{R} - W^{R} H^{R} } \right\|_{F}^{2} }}\;\;\;\;\lambda_{2} = \frac{{\left\| {X^{L} - W^{L} H^{L} } \right\|_{F}^{2} }}{{2tr((W^{L} )^{T} AW^{R} )}}\;\;\;\;\;\mu = \frac{{\left\| {X^{L} - W^{L} H^{L} } \right\|_{F}^{2} }}{{2(\left\| {W^{L} } \right\|_{F}^{2} + \left\| {W^{R} } \right\|_{F}^{2} )}} $$

The scaling of these parameters is controlled by a set of multipliers. In all data used in this paper, the multiplier of $\lambda_{1}$, $\lambda_{2}$ and $\mu$ are 1, 1000 and 1, respectively.

Choosing parameter $K$, that is, evaluating the number of CSCPs, is a difficult issue. When the number of cell subtypes within a cell type is unknown, we recommend choosing $K$ as small as possible, that is, $K$ = 2 or 3. For Single-cell transcriptome sequencing data of cancer immunotherapy patients, since the numbers of CD8 + T cell subtypes and macrophage subtypes are known, we choose the average of number of these two subtypes, that is, $K$ = 7.

### Statistical test

Wilcoxon signed-rank test is used to test differences in cell distances and Jaccard coefficients, which are implemented by the ‘wilcoxon’ function (with default parameters) in the Python ‘scipy’ package.

T test is used to test differences in normalized gene expression, which are implemented by the ‘ttest_ind’ function (with default parameters) in the Python ‘scipy’ package.

### Ligand–receptor information

We collect 2557 human ligand–receptor pairs from FANTOM5^67^ and convert them to 2324 mouse ligand–receptor pairs through the human-mouse homologous gene database^68^.

### Single cell spatial transcriptome sequencing data

We collect sequential fluorescence in situ hybridization (seqFISH+) data of mouse visual cortex and olfactory bulb, respectively^69^. Each FOV covers the following cell types: astrocytes, microglia cell, endothelial cell, oligodendrocytes and oligodendrocyte precursor cells (OPCs), which are known to communicate with each other^26–34^. These 5 cell types are paired in each FOV, resulting in ${\rm A}_{5}^{2}$ = 20 cell type pairs per FOV (e.g. Astro → Micro, Astro → Endo, Astro → Oligo, Astro → OPC, Micro → Astro and Micro → Endo, etc.). Cell types with less than 5 cells are removed in each FOV, remaining 12 pairs of cell types on olfactory bulb (4 FOVs) and 44 pairs of cell types on visual cortex (6 FOVs). We use Seurat R package to preprocess the data with default parameters^70^.

### Single-cell transcriptome sequencing data for generating sampled data

We collect scRNA-seq data on cerebral cortex of four mice (two male and two female)^35^. Each data cover four cell types which are known to communicate with each other, including astrocytes, endothelial, microglia and oligodendrocytes. These 4 cell types are paired for each mouse, resulting in ${\rm A}_{4}^{2}$ = 12 cell type pairs for each mouse. We use Seurat R package to preprocess the data with default parameters^70^.

### Single-cell transcriptome sequencing data of cancer immunotherapy patients

We collect scRNA-seq data of 29 breast cancer patients before anti-PD-1 therapy^42^, including 9 patients responded to immunotherapy (expansion of immune cells after immunotherapy) and 20 patients did not (non-expansion). We use Seurat R package to preprocess the data with default parameters^70^.

### Cell–cell communication methods

We collect 13 state-of-art cell–cell communication methods according to the following criteria: (1) single cell transcriptome data, paired cell type annotations and known ligand–receptor gene pairs as inputs and (2) communication scores or significance of ligand–receptor pairs as outputs. Given cell type A (sender cell type) and cell type B (receiver cell type), the details of these methods are as follows:

#### CellCall^71^

The calculation of the communication score for each ligand–receptor gene pair is the sum of two parts, the first part is the sum of the mean expression values of the ligand in cell type A and that of the receptor in cell type B, the second part is the enrichment score of the downstream genes of the receptor gene by gene set enrichment analysis. We consider ligand–receptor pairs under default parameters for subsequent analysis.

#### CellChat^5^

The communication probability is calculated based on law of mass action, considering the geometric means of ligand in cell type A and the receptor in the cell type B and evaluating the statistical significance of the communication by permutation test. All ligand–receptor pairs inferred under default parameters are selected for our subsequent analysis.

#### CellPhoneDB^4^

The communication score is calculated by averaging the means expression of ligand in cell type A and that of the receptor in the cell type B and obtained the statistical significance of the communication score by permutation test. The ligand–receptor pairs with P-value < 0.05 are selected for our subsequent analysis.

#### CytoTalk^6^

The signaling network between cell type A and B is constructed by weighting the mutual information of gene–gene interactions (including ligand–receptor interactions) and predicting inter- and intracellular signaling pathways by prize-collecting Steiner Forest algorithm. We select ligand–receptor pairs under default parameters for subsequent analysis.

#### ICELLNET^72^

The communication score is calculated by multiplying the geometric means of ligand and receptor expressions in cell type A and B, respectively. The ligand–receptor pairs under default parameters are selected for our subsequent analysis.

#### Kumar^73^

The communication score is the product of the mean ligand expression in cell type A and the mean receptor expression in the cell type B. The ligand–receptor pairs with score > 0 are considered for our subsequent analysis.

#### NATMI^74^

The communication score is calculated by multiplying the means of ligand and receptor expressions in cell type A and B, respectively. We select ligand–receptor pairs under default parameters are selected for subsequent analysis.

#### NicheNet^57^

The communication score (i.e. ligand-target signaling importance) is calculated by personalized PageRank according to the means of ligand and receptor expressions in cell type A and B, respectively. The ligand–receptor pairs under default parameters are selected for our subsequent analysis.

#### SingleCellSignalR^75^

The nonlinear function is designed to scoring communication probabilities based on the product of ligand and receptor expression in cell type A and B. The ligand–receptor pairs under default parameters are considered for our subsequent analysis.

#### Skelly^73^

The ligand–receptor pairs are considered to be involved in cell–cell communication when ligand and receptor are expressed in more than 20% of cells in cell type A and B, respectively.

#### iTALK^76^

The ligand–receptor pairs are selected by selecting ligand and receptor that is differentially expressed in cell type A and B, respectively. The ligand–receptor pairs under default parameters are selected for our subsequent analysis.

#### scConnect^77^

The communication score of ligand–receptor pairs is calculated as the geometric-mean of the sending score and the receiving score, according to the means of ligand and receptor expressions in cell type A and B, respectively. We consider ligand–receptor pairs under default parameters for subsequent analysis.

#### scMLnet^7^

A multilayer network is constructed based on ligands, receptors, and target genes, and the sub-network is refined by selecting edges with significant positive correlation. The ligand–receptor pairs under default parameters are considered for our subsequent analysis.

### Classification of breast cancer patient with anti-PD-1 therapy

We randomly select 4/5 of the patients for training (i.e. 16 non-responding patients and 7 responding patients) and use the remaining 1/5 of the patients for testing (i.e. 4 non-responding patients and 2 responding patients). We train and test the SVM 100 times with CSCP-based features and cell-type-based features, and finally present the average of 100 results.

We do not keep sample balanced for two reasons. Firstly, only a small number of patients respond to anti-PD1 therapy. If the number of non-responsive and responding patients is balanced, the model could not be applicable to the real situation. Second, according to the description of unbalanced data by Van et al. and Cieslak et al., unbalanced datasets often have much fewer samples in some categories than in another^78,79^. In our dataset, the ratio of number of non-responding patients and responding patients is close to 2:1 and the phenomenon of data imbalance is not obvious.

### Supplementary Information

Supplementary Figures.

## Data Availability

The source codes are available at https://github.com/GaoLabXDU/cell-sub-crosstalk-pair. The seqFISH + data of mouse visual cortex and olfactory bulb is downloaded from https://github.com/CaiGroup/seqFISH-PLUS. The scRNA-seq data of mice cerebral cortex is downloaded from https://singlecell.broadinstitute.org/single_cell/study/SCP795. The scRNA-seq data of breast cancer patients are downloaded from https://lambrechtslab.sites.vib.be/en/single-cell.
