# Supplementary material for: Defining and identifying cell sub-crosstalk pairs for characterizing cell–cell communication patterns
Source: Sci Rep. 2023 Sep 21;13:15746. doi: 10.1038/s41598-023-42883-8 (PMC10514069; doi:10.1038/s41598-023-42883-8)
Supplement: Supplementary file 1 — Supplementary Figures. [file 41598_2023_42883_MOESM1_ESM.pdf]

Defining and identifying cell sub-crosstalk pairs for characterizing cell-cell communication patterns

Chenxing Zhang[1], Yuxuan Hu[1], Lin Gao[1,*]

1 School of Computer Science and Technology, Xidian University, Xi'an, China

Corresponding Author:

Lin Gao, School of Computer Science and Technology, Xidian University, 710071, Xi'an, China. Tel.: +86-29-88202354; Email: lgao@mail.xidian.edu.cn

Supplementary Figures

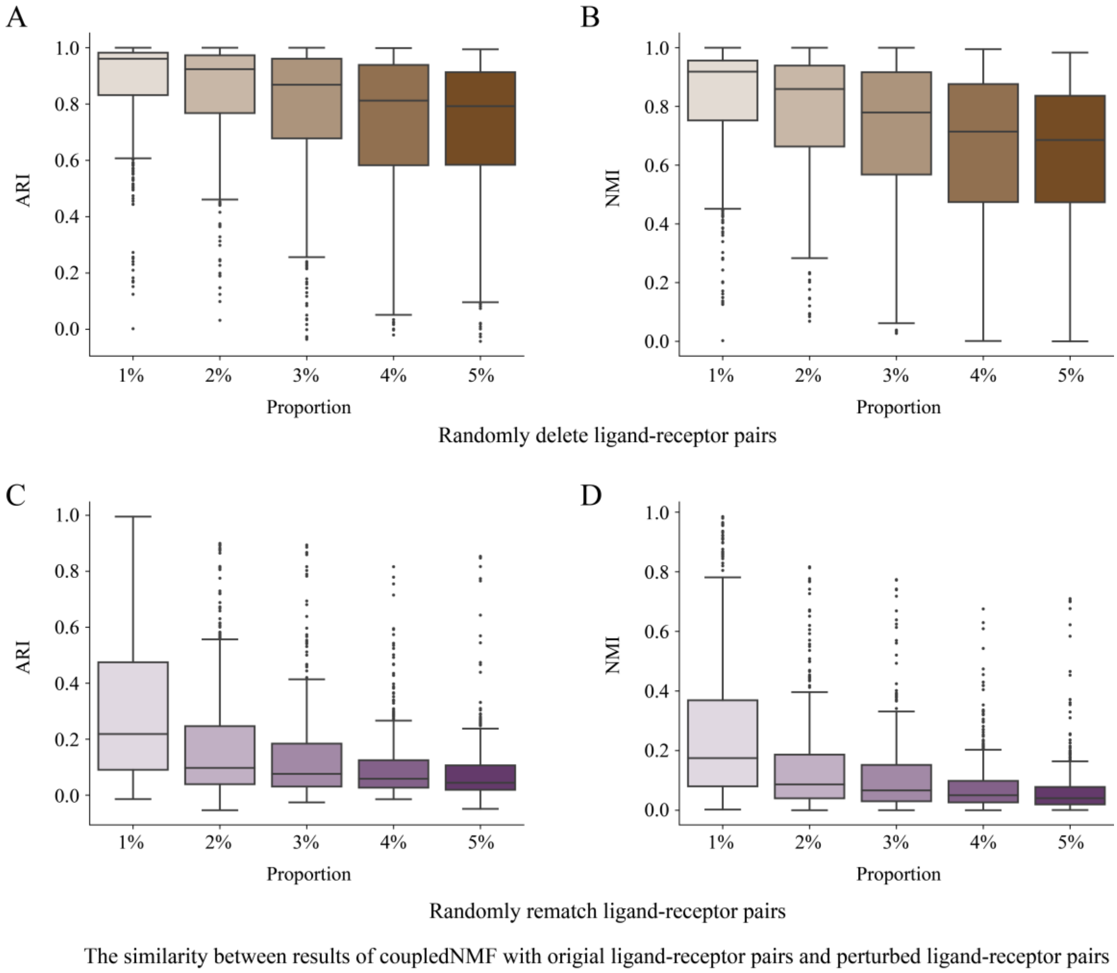

Fig S1. The sensitivity of ligand-receptor information. The box plot shows the similarity (quantified by ARI or NMI) between CSCPs based on the original ligand-receptor pairs and (A) (B) randomly deleted ligand-receptor pairs at different proportions or (C) (D) randomly rematched ligand-receptor pairs at different proportions. ARI represents adjusted Rand index and NMI represents normalized mutual information.

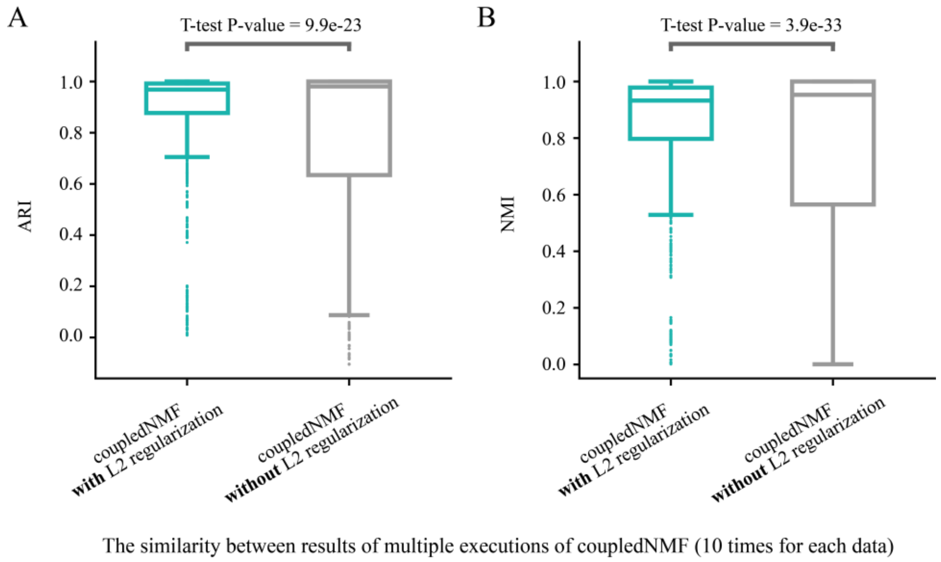

Fig S2. The stability comparison of coupled NMF with and without L2 regularization. The box plot shows the similarity between the results of multiple executions of coupled NMF (10 times for each dataset) is quantified using (A) Adjusted Rand Index (ARI) and (B) Normalized Mutual Information (NMI). The green box represents the similarity based on coupled NMF with L2 regularization, while the grey box represents similarity without L2 regularization.

A

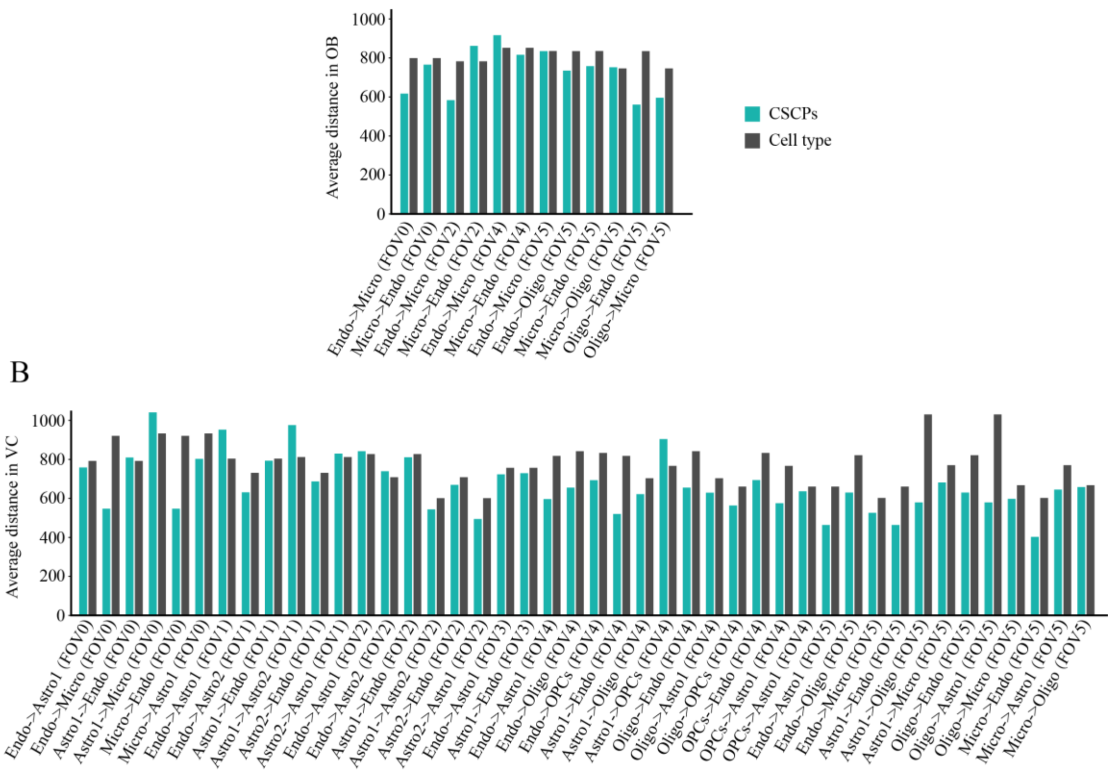

Fig S3. The average distance between cells in CSCPs and cell types for each cell type pair. (A) the average distance in olfactory bulb. (B) the average distance in visual cortex. The green bar represents the average distance of cells within CSCPs. The grey bar represents the average distance of cells within cell types.

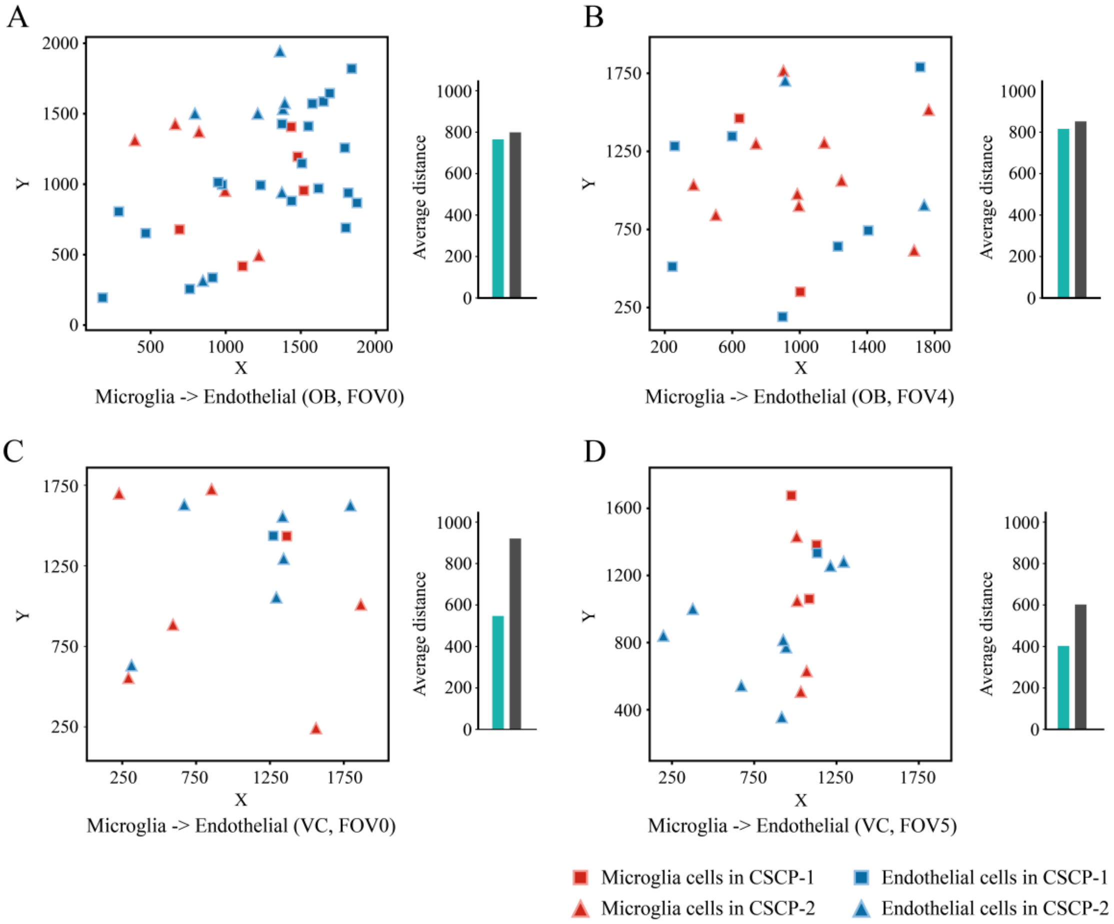

Fig S4. Visualization of cell spatial location and average distance between microglia cells and endothelial cells in (A) olfactory bulb, FOV0, (B) olfactory bulb, FOV4, (C) visual cortex, FOV0 and (D) visual cortex, FOV5. The red squares represent the sender cells in CSCP-1, the blue squares represent the receiver cells in CSCP-1, the red triangles represent the sender cells in CSCP-2, the blue triangles represent the receiver cells in CSCP-2. The grey bar represents the average distance of cells within cell types. (OB, olfactory bulb; VC, visual cortex)

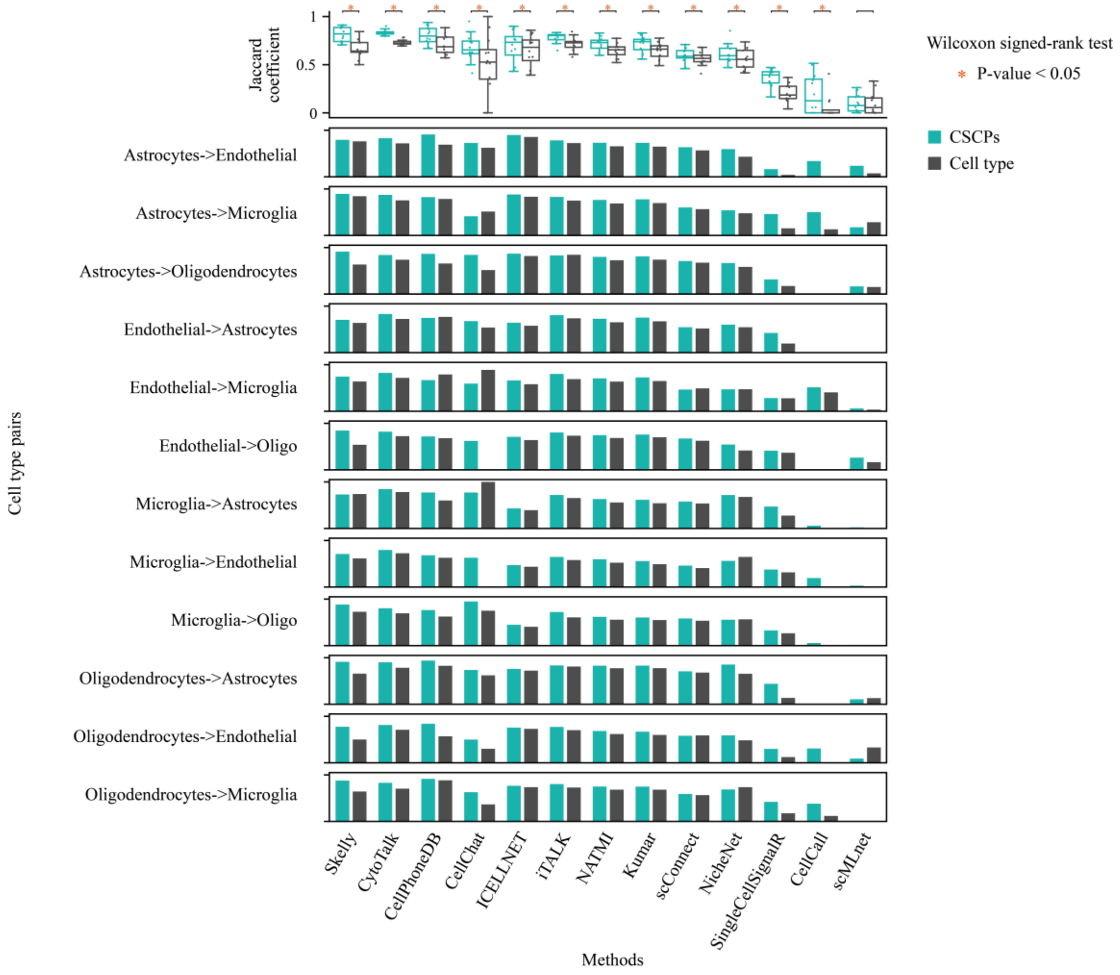

Fig S5. The Jaccard coefficient of 13 cell-cell communication methods and 12 cell type pairs in mouse M001. The top section displays the box plot of Jaccard coefficient for each cell-cell communication methods. The bottom section displays the bar plot of Jaccard coefficient for each cell-cell communication methods in each cell type pairs.

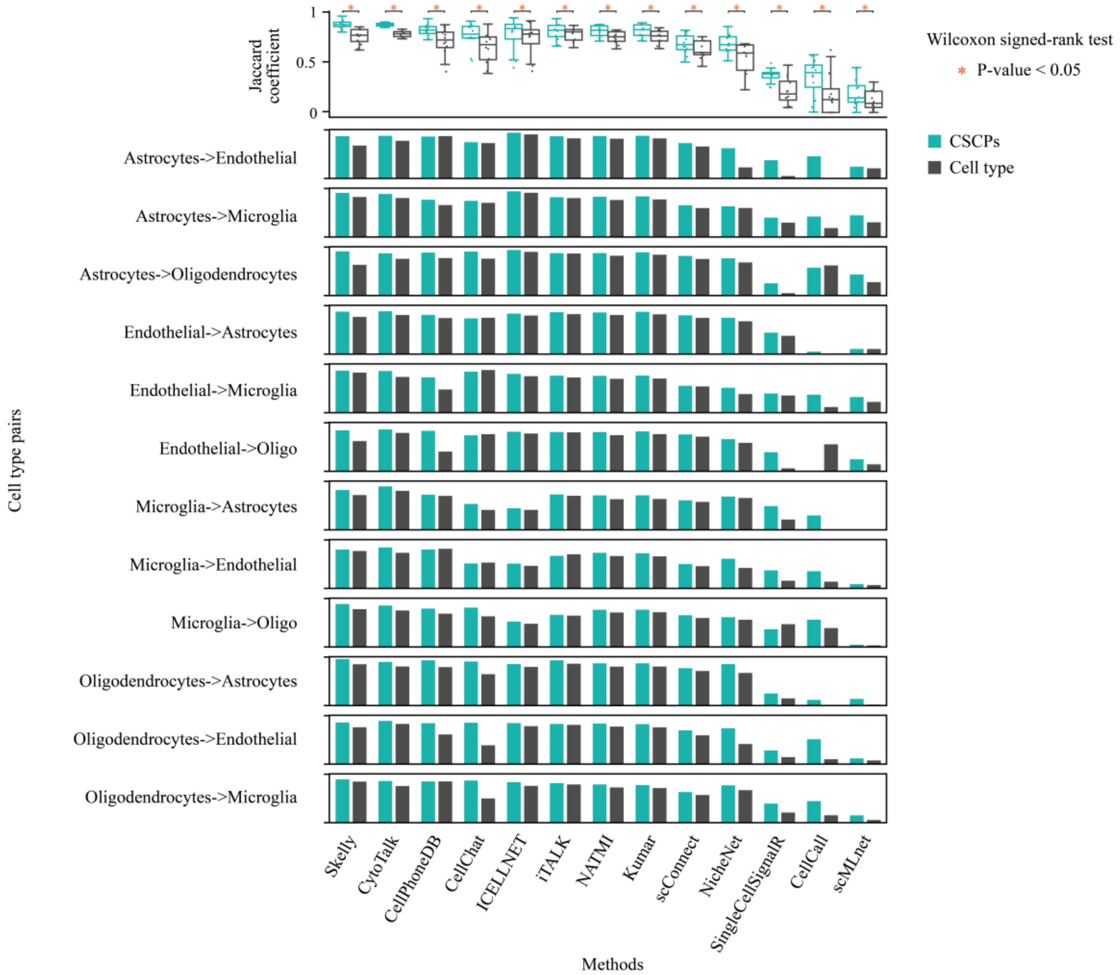

Fig S6. The Jaccard coefficient of 13 cell-cell communication methods and 12 cell type pairs in mouse M002. The top section displays the box plot of Jaccard coefficient for each cell-cell communication methods. The bottom section displays the bar plot of Jaccard coefficient for each cell-cell communication methods in each cell type pairs.

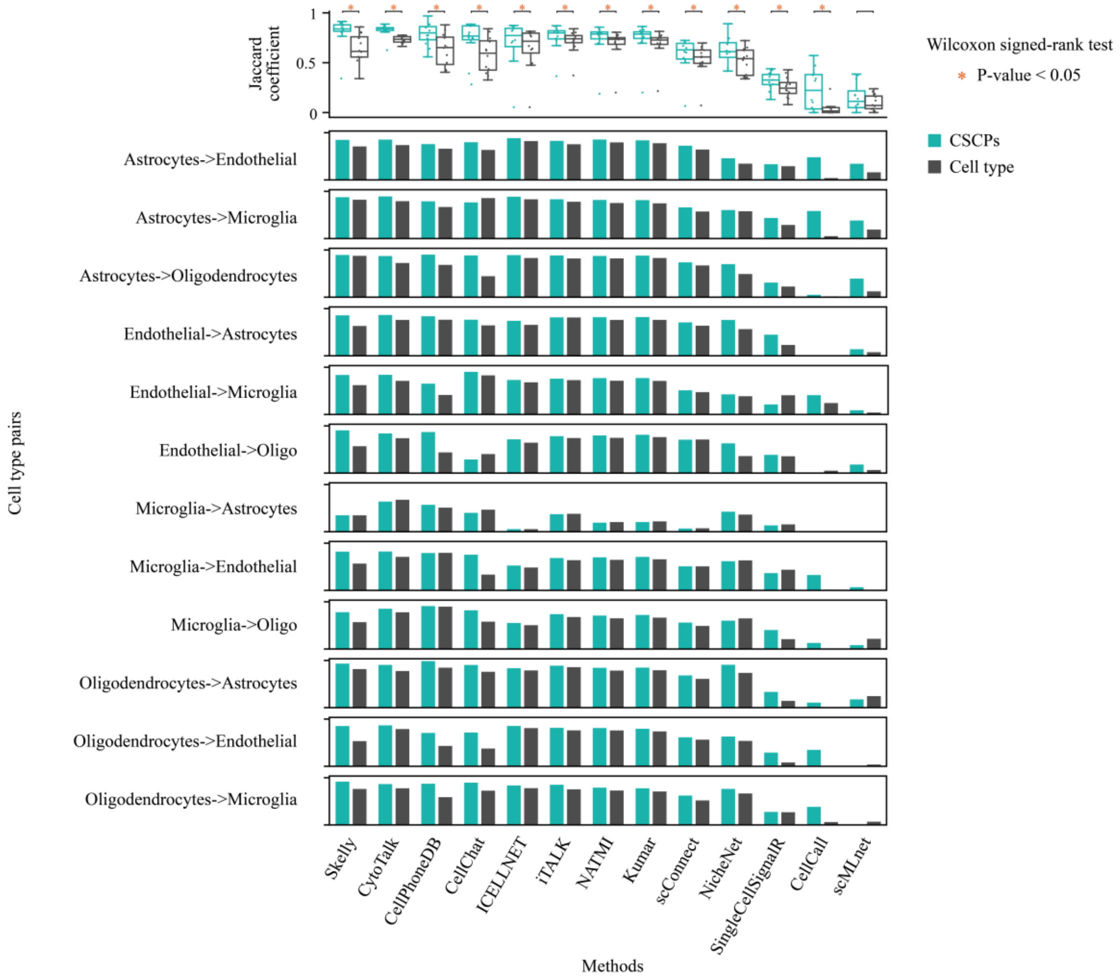

Fig S7. The Jaccard coefficient of 13 cell-cell communication methods and 12 cell type pairs in mouse F001. The top section displays the box plot of Jaccard coefficient for each cell-cell communication methods. The bottom section displays the bar plot of Jaccard coefficient for each cell-cell communication methods in each cell type pairs.

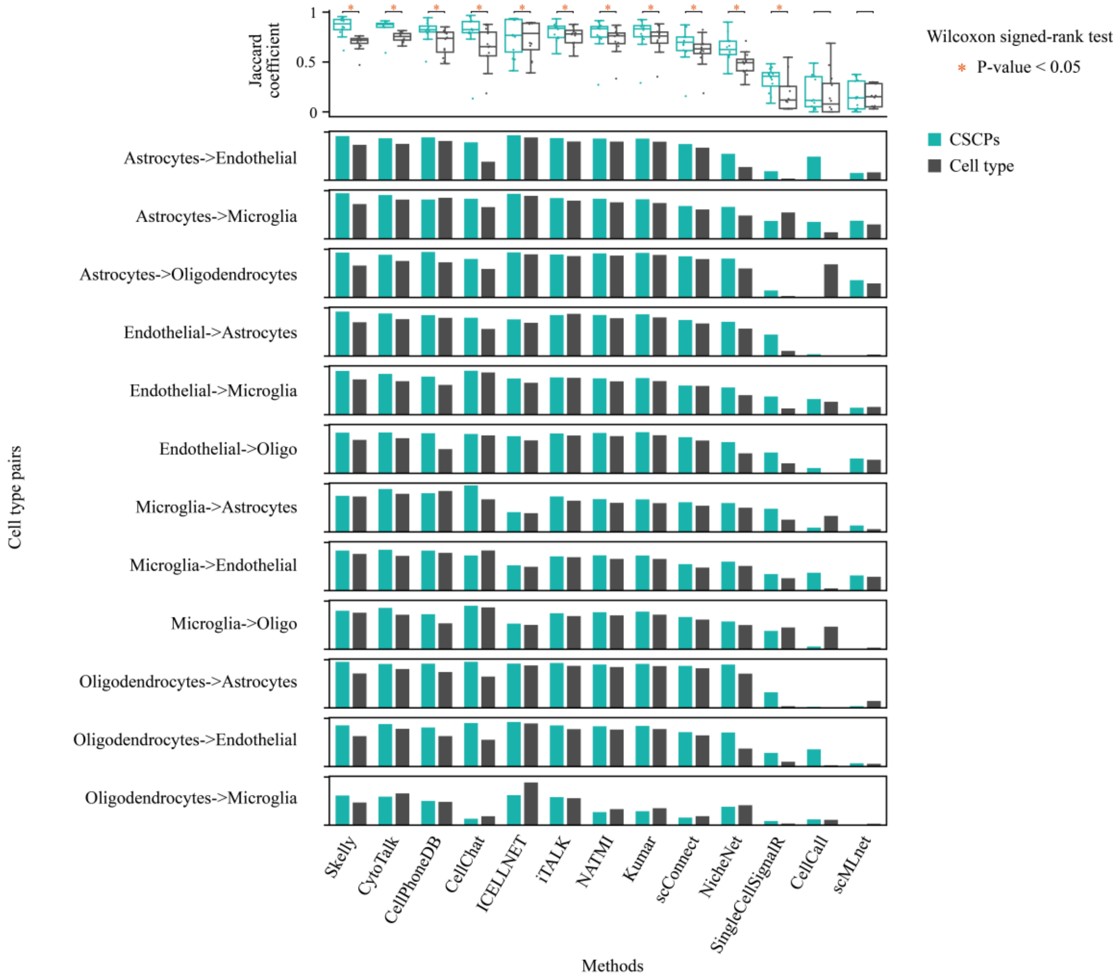

Fig S8. The Jaccard coefficient of 13 cell-cell communication methods and 12 cell type pairs in mouse F002. The top section displays the box plot of Jaccard coefficient for each cell-cell communication methods. The bottom section displays the bar plot of Jaccard coefficient for each cell-cell communication methods in each cell type pairs.

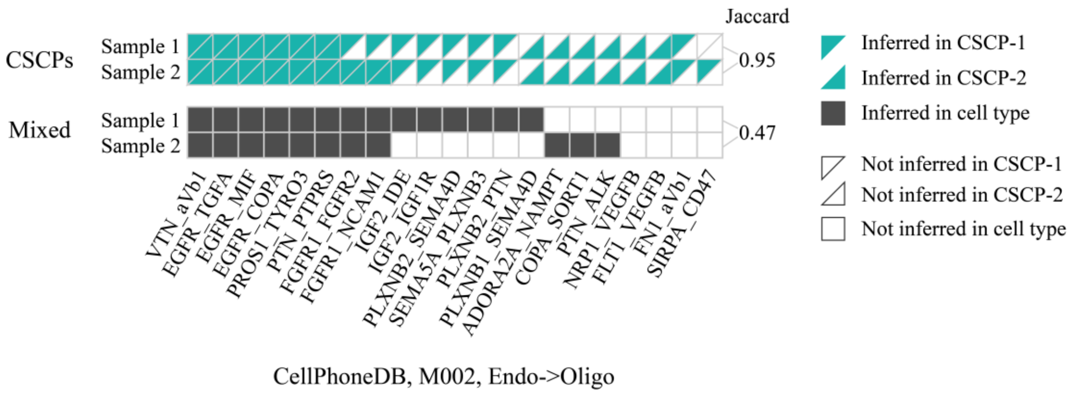

Fig S9. The visualization of ligand-receptor pairs inferred by CellPhoneDB between endothelial cells and astrocytes cells. The green triangles indicate inferred ligand-receptor pairs in CSCPs (top section) and the black squares indicate inferred ligand-receptor pairs in cell types (bottom section).

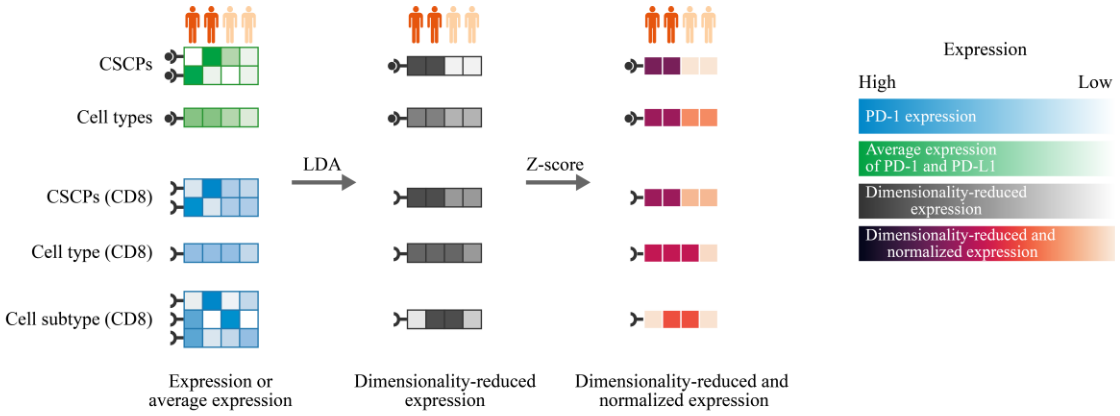

Fig S10. The diagram of dimension reduction and normalization of expression and average expression. The expression of cell subtype and CSCPs for each patient is condensed into a single dimension using linear dimensionality reduction (LDA). Subsequently, z-score normalization is applied to the one-dimensional expression of cell type, cell subtype, and CSCPs for all patients individually.

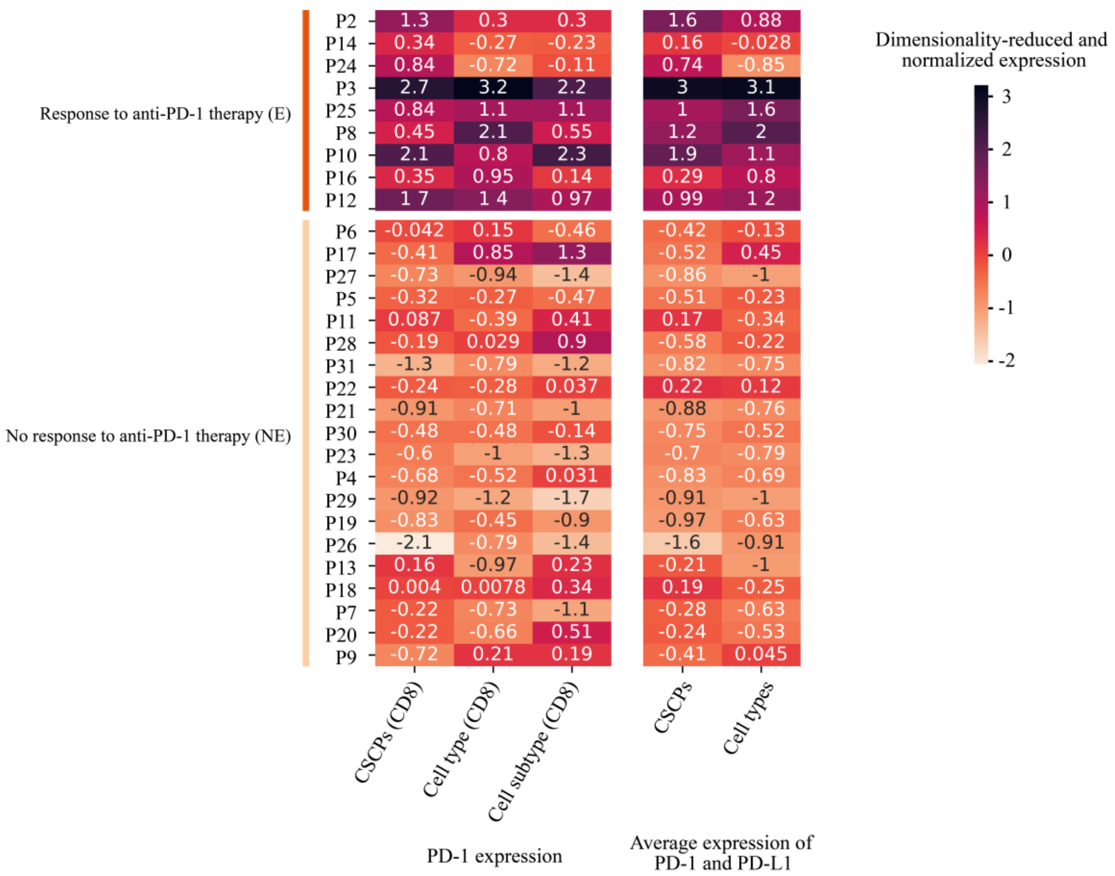

Fig S11. The dimensionality-reduced and normalized expression of each feature for each patient. The top left section of heatmap shows the expression of PD-1 in CD8+ T cells from patients who respond to anti-PD-1 therapy. The bottom left section shows the expression of PD-1 in CD8+ T cells from patients who do not respond to therapy. The top right section shows the average expression of PD-L1 and PD-1 in macrophages and CD8+ T cells from patients who respond to therapy. The bottom right section shows the average expression of PD-L1 and PD-1 in macrophages and CD8+ T cells from patients who do not respond to therapy.

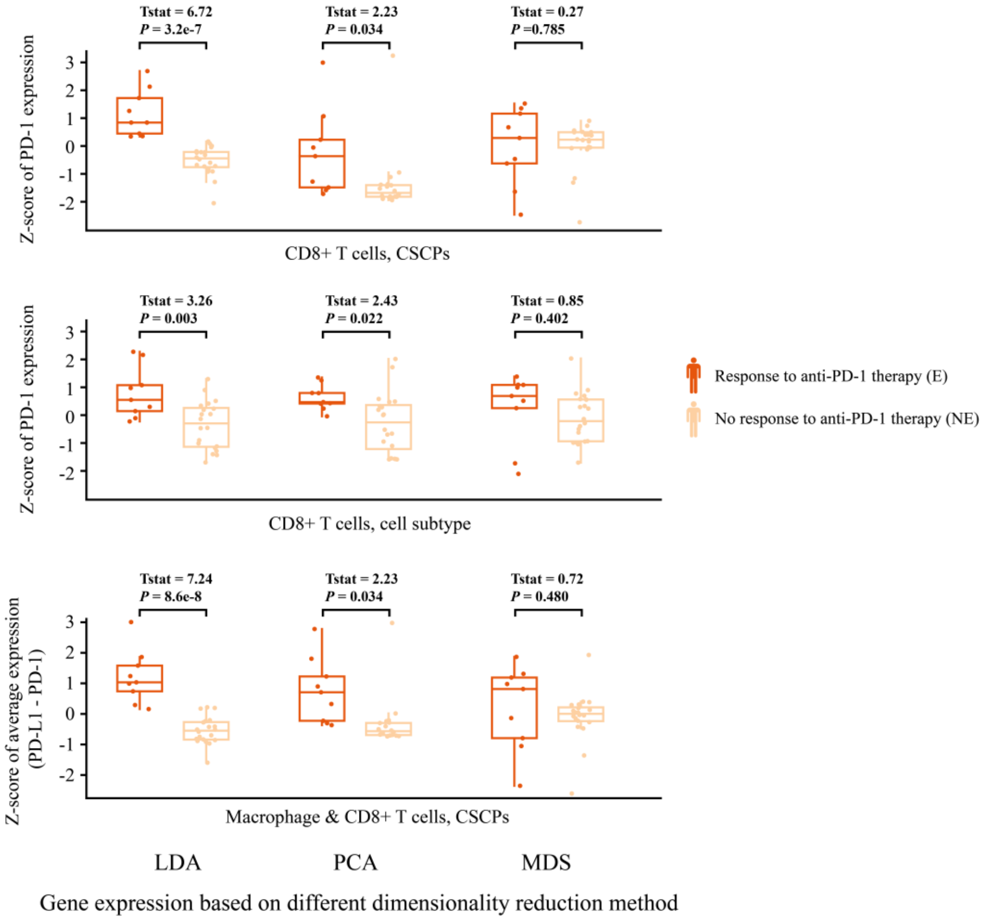

Fig S12. Comparison of gene expression based on different dimensionality reduction method. The box plot shows the z-scores of PD-1 expression in CSCPs (top section), z-scores of PD-1 expression in cell subtypes (middle section), and z-scores of the average expression of PD-L1 and PD-1 in CSCPs (bottom section) after different dimension reduction techniques, including linear discriminant analysis (LDA), principal component analysis (PCA), and multidimensional scaling (MDS). The dark orange color represents response to anti-PD-1 therapy, while the light orange color represents no response to anti-PD-1 therapy.
